# The complex pattern of epigenomic variation between natural yeast strains at single-nucleosome resolution

**DOI:** 10.1186/s13072-015-0019-3

**Published:** 2015-07-31

**Authors:** Fabien Filleton, Florent Chuffart, Muniyandi Nagarajan, Hélène Bottin-Duplus, Gaël Yvert

**Affiliations:** Laboratoire de Biologie Moléculaire de la Cellule, Ecole Normale Supérieure de Lyon, CNRS, Université de Lyon, 46 Allée d’Italie, 69007 Lyon, France; Department of Genomic Science, School of Biological Sciences, Central University of Kerala, Kerala, India

**Keywords:** Epigenomics, Histone modification, Yeast, Evolution, Natural strains, Epi-polymorphism, Epi-allele, Ecology

## Abstract

**Background:**

Epigenomic studies on humans and model species have revealed substantial inter-individual variation in histone modification profiles. However, the pattern of this variation has not been precisely characterized, particularly regarding which genomic features are enriched for variability and whether distinct histone marks co-vary synergistically. Yeast allows us to investigate intra-species variation at high resolution while avoiding other sources of variation, such as cell type or subtype.

**Results:**

We profiled histone marks H3K4me3, H3K9ac, H3K14ac, H4K12ac and H3K4me1 in three unrelated wild strains of *Saccharomyces cerevisiae* at single-nucleosome resolution and analyzed inter-strain differences statistically. All five marks varied significantly at specific loci, but to different extents. The number of nucleosomes varying for a given mark between two strains ranged from 20 to several thousands; +1 nucleosomes were significantly less subject to variation. Genes with highly evolvable or responsive expression showed higher variability; however, the variation pattern could not be explained by known transcriptional differences between the strains. Synergistic variation of distinct marks was not systematic, with surprising differences between functionally related H3K9ac and H3K14ac. Interestingly, H3K14ac differences that persisted through transient hyperacetylation were supported by H3K4me3 differences, suggesting stabilization via cross talk.

**Conclusions:**

Quantitative variation of histone marks among *S. cerevisiae* strains is abundant and complex. Its relation to functional characteristics is modular and seems modest, with partial association with gene expression divergences, differences between functionally related marks and partial co-variation between marks that may confer stability. Thus, the specific context of studies, such as which precise marks, individuals and genomic loci are investigated, is primordial in population epigenomics studies. The complexity found in this pilot survey in yeast suggests that high complexity can be anticipated among higher eukaryotes, including humans.

**Electronic supplementary material:**

The online version of this article (doi:10.1186/s13072-015-0019-3) contains supplementary material, which is available to authorized users.

## Background

Epigenomes differ between individuals and the extent of this diversity has received increasing attention because of its potential impact on cellular processes (proliferation, differentiation, response to environmental cues and contribution to pathological processes). For various species, from yeast to plants and mammals, natural populations were found to display substantial variation in several chromatin hallmarks, including DNA methylation [1–5], histone post-translational modifications [6–9] and accessibility to DNA [10, 11]. However, registering this diversity and understanding its possible consequence is challenging, because variation in chromatin marks has complex properties. Unlike the DNA sequence itself, chromatin modifications include a large repertoire of chemical modifications. These modifications are reversible, they are established and removed dynamically at various timescales, they differ between cell types and their presence or absence can result from a response to environmental conditions. In addition, several cross talk mechanisms have been described, where the presence of one modification aided the establishment of other modifications. Comparing epigenomes is therefore more challenging than population genomics, because there is more to read than a four nucleotide sequence and the ‘text’ differs quantitatively rather than qualitatively, not only between individuals, but also according to various factors. In this regard, yeast offers the possibility to study epigenomic variation in a simplified context, where the complexity of multiple cell types is avoided and environmental conditions can be controlled.

Although chromatin marks are often called ‘epigenetic marks’, we have avoided this term here because their mode of inheritance is not the focus of the study. We nonetheless use the term ‘epigenome’ to describe the genomic profile of one or several chromatin marks, and the term ‘epi-polymorphism’ to describe variation in the epigenome, regardless of the possible epigenetic maintenance of these marks.

Histone post-translational modifications comprise a large set of chromatin marks, and their variation within species has been investigated in several studies. In yeast, about 10% of nucleosomes displayed quantitative differences in H3K14 acetylation between two wild strains [6]. In *A. thaliana*, the genomic profiles of H3K4me2 and H3K27me3 were reported for the Col-0 and Cvi accessions [12]. The authors compared the lists of regions associated with H3K4me2 in the two accessions, which differed for about 4% of genic regions and 25% of transposable element regions. Variation was also seen for H3K27me3-associated regions, with 10% of genic regions and 20% of transposable element regions specifically listed in either Col-0 or Cvi. These proportions are comparable to the fraction of the rat genome subjected to variation in H3K4me1, H3K4me3, H3K27me3 and H4K20me1 between strains. These marks were profiled recently in liver and heart tissues using a statistical design that revealed quantitative differences in 7–16% of the regions tested [13]. In humans, inter-individual variations in histone modifications have been estimated from lymphoblastoid cell lines. An initial study detected allelic specificities and Mendelian inheritance of histone acetylation in families of the Human Polymorphism Study Center [14]. A more recent characterization of human lymphoblastoid cells revealed that the proportion of allele-specific sites ranged from 6% (for H4K20me1) to 30% (for H3K27me3) [7]. In a parallel study, between 10% (H3K36me3) and >30% (H3K27ac) of genomic regions were reported to vary between cell lines derived from different individuals [9]. Taken together, these studies illustrate that histone modification marks vary within species at a substantial fraction of genomic positions.

Histone modifications are closely associated with transcriptional regulations; therefore, their variation could reflect variation in transcript levels. This proved to be only partially true. In human lymphoblastoid cell lines, Kasowski et al. reported that genes showing histone marks variability at multiple enhancers tended to vary in expression as well [8]. However, 74% of genes with no expression variation also displayed variable levels of histone modification at one or more enhancers. Kilpinen et al. reported a significant haplotypic coordination between transcription levels and H3K27ac, H3K4me1 and H3K4me3 variation across gene regions, but no association with H3K27me3 variation [7]. McVicker et al. reported that expression quantitative trait locus (eQTL) alleles enhancing transcription are associated with lower levels of H3K27me3 and higher levels of H3K4me3 and H3K27ac around the transcription start site (TSS) [9]. These observations are consistent with those from Rintisch et al. who found that 14–20% of eQTLs in rat tissues were also QTLs of histone modifications (and vice versa). However, 36% of genes displayed variable histone marks that could not be attributed to transcriptional differences [13]. This is also in line with previous observations in yeast, where H3K14ac variation was not systematically associated with differences in mRNA levels [6] and where ≥30% of QTLs of H3K14ac could not be attributed to genetic regulations of gene expression [15]. These studies demonstrated the link between variation in histone marks and variation in gene expression, but also showed that this association is not systematic. It would be informative to describe which genomic regions support this association in a given organism and tissue.

Another important question is whether different histone marks co-vary between individuals. Co-variation of distinct marks has been observed, but not as a systematic correlation. At the scale of entire human genes, correlated allelic imbalances were reported between distinct histone marks [7]. Co-variation among active marks was remarkably apparent when considering only the genomic regions where DNase-sensitivity was under genetic control [9]. However, the expected negative correlation with the repressive mark H3K27me3 was not observed [7] or only poorly [9]. In rat tissues, numerous QTLs were found to control both H3K4me3 and H3K27me3, but 25% of these co-regulations acted in a compensating manner (e.g., upregulating H3K27me3 together with H3K4me3, which is expected to act antagonistically on gene expression) [13]. These observations demonstrated the existence of correlated patterns of variation. However, the extent and location of co-variation between marks remain poorly characterized.

In the present study, to obtain a detailed view of intra-species variation in histone modifications, we determined the quantitative variation of five marks at single-nucleosome resolution in *Saccharomyces cerevisiae*. We took advantage of three wild strains for which transcriptome variation has been extensively characterized. This new dataset allowed us to precisely map the location of variation, to investigate whether variability is focused on precise nucleosomes or spread on many consecutive ones and to determine the degree of co-variation between marks on individual nucleosomes.

## Results and discussion

We report on the extent and pattern of intra-species quantitative variation of histone modifications in the epigenome of *S. cerevisiae*. Three natural yeast strains were compared. For each strain, epigenomic profiling of five histone modification marks was performed at single-nucleosome resolution, in biological replicates. Evidently, profiling more strains would provide a larger view of variation, but we invested in biological replicates instead to control for biological and technical variability. In this section, we describe and discuss results first at the genomic scale, then at the level of protein-coding genes and finally at the level of individual nucleosomes.

### Wild yeast strains display distinct histone modification epigenomes

Our previous observation of H3K14 acetylation variation between two natural strains [6, 15] raised questions regarding specificity: Is this variation caused by one strain having a specific H3K14ac profile? Do nucleosomes displaying H3K14ac variation also vary in their level of other chromatin marks? To address these questions, we chose to profile the epigenome of this and four other histone modifications, in three strains. H3K14, H3K9 and H4K12 acetylation, as well as H3K4 tri-methylation are abundant in highly transcribed genes and are particularly present in the 5′ parts of gene bodies [16–18]. These four marks therefore target many nucleosomes in common. Studying them simultaneously in three strains should determine whether variation of H3K14ac correlates with the variation of other marks. In addition, we chose to profile H3K4 mono-methylation because it is distributed on a different set of nucleosomes: those located at the 3′ of gene bodies [16, 18]. This way, all five marks cover biochemical information on the majority of the chromatin. Such a dataset should also reveal if one strain is particularly different from the two others. The strains used were BY, RM and YJM789 (hereafter called ‘YJM’). BY and RM are those where H3K14ac was studied previously [6]. YJM derives from a clinical isolate and is genetically equidistant to BY and RM, with an SNP frequency of about 0.5% [19, 20]. All three strains have been used extensively to study complex traits and to map genetic determinants of gene expression variation; their genomes have been fully sequenced and assembled [19, 21–23].

We first verified the specificity of antibodies by Western blot, by probing extracts of yeast strains carrying point mutations in histones [24] (Additional file 1A). We also compared on Western blot the three strains, BY, RM and YJM, for their level of bulk histone acetylation or methylation and saw no sign of global differences (Additional file 1B).

We then grew each strain in 18 independent cultures, using standard laboratory conditions. For each strain, three samples were processed for MNase-seq to identify nucleosome positions. All other samples were processed for MN-ChIP-seq (immunoprecipitation of chromatin after MNase digestion, followed by sequencing). The design was such that every antibody was applied on three biological replicates of each strain, except for five samples which did not meet the quality criteria (see “Methods”). The experiment produced between 7 and 41 million Illumina short reads per sample (Additional file 2). Reads were then mapped to the genome of the relevant strain. To enable quantitative comparisons between the strains, we performed pairwise alignments of their genomes, producing tables of corresponding coordinates. We defined regions of sufficient sequence similarity for unambiguous alignment (called common uninterrupted regions or CURs, see “Methods”), which altogether covered ~8.5 Mb of the 12 Mb genome. Note that regions of high sequence divergence (such as transposons) were excluded from our analysis, because they are not amenable to quantitative comparisons of MNase-based ChIP-seq data. The diversity reported hereafter therefore likely reflects a low boundary of the whole epigenomic variation.

**Fig. 1:**
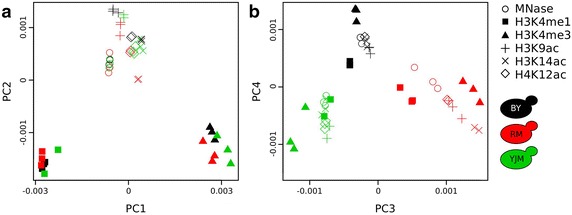
Principal component analysis (PCA) of epigenomic variation. Each *dot* represents one experiment, with *symbols* indicating the antibodies that were used (if any) and *colors* indicating the strains. PCA was performed on genomic coverages by dividing the genome into 90 bp bins and counting the number (per million) of forward sequence reads covering each bin. **a** The first two components discriminate the nucleosomal marks (*identical symbols* are grouped). **b** The next two components discriminate the strains (*identical colors* are grouped).

To visualize how samples differed from one another, we performed a principal component analysis (PCA) of the genome coverage profiles. The first four principal components were statistically significant, as determined by a permutation test, and explained 78% of the variance in the data (Additional file 3). We plotted the samples according to their coordinates along these components. Remarkably, the first two components discriminated histone marks, showing that the profile of each mark was specific and globally consistent across the strains (Fig. 1a). Samples corresponding to the three acetylation marks tended to group together. This was expected given the similar distribution of these marks along gene bodies. They were also proximal to the MNase-seq samples, which is consistent with the presence of these acetylation marks on many nucleosomes. H3K4me1 profiles differed from all others, which was expected because this mark targets a distinct set of nucleosomes. Finally, H3K4me3 also defined its own group, which probably reflected its known enrichment in small regions containing TSSs. Strikingly, components 3 and 4 discriminated the strains, which revealed the presence of intra-species variation for all marks (Fig. 1b). Importantly, biological replicates were grouped together, showing that inter-strain variation was greater than intra-strain biological and technical variability. If PCA was applied on ChIP-seq profiles normalized by the mean MNase-seq profile of each strain, marks were again strongly discriminated by (PC1, PC2) and strains were separated by (PC3, PC4), but, as expected, less strongly (Additional file 4).

**Fig. 2:**
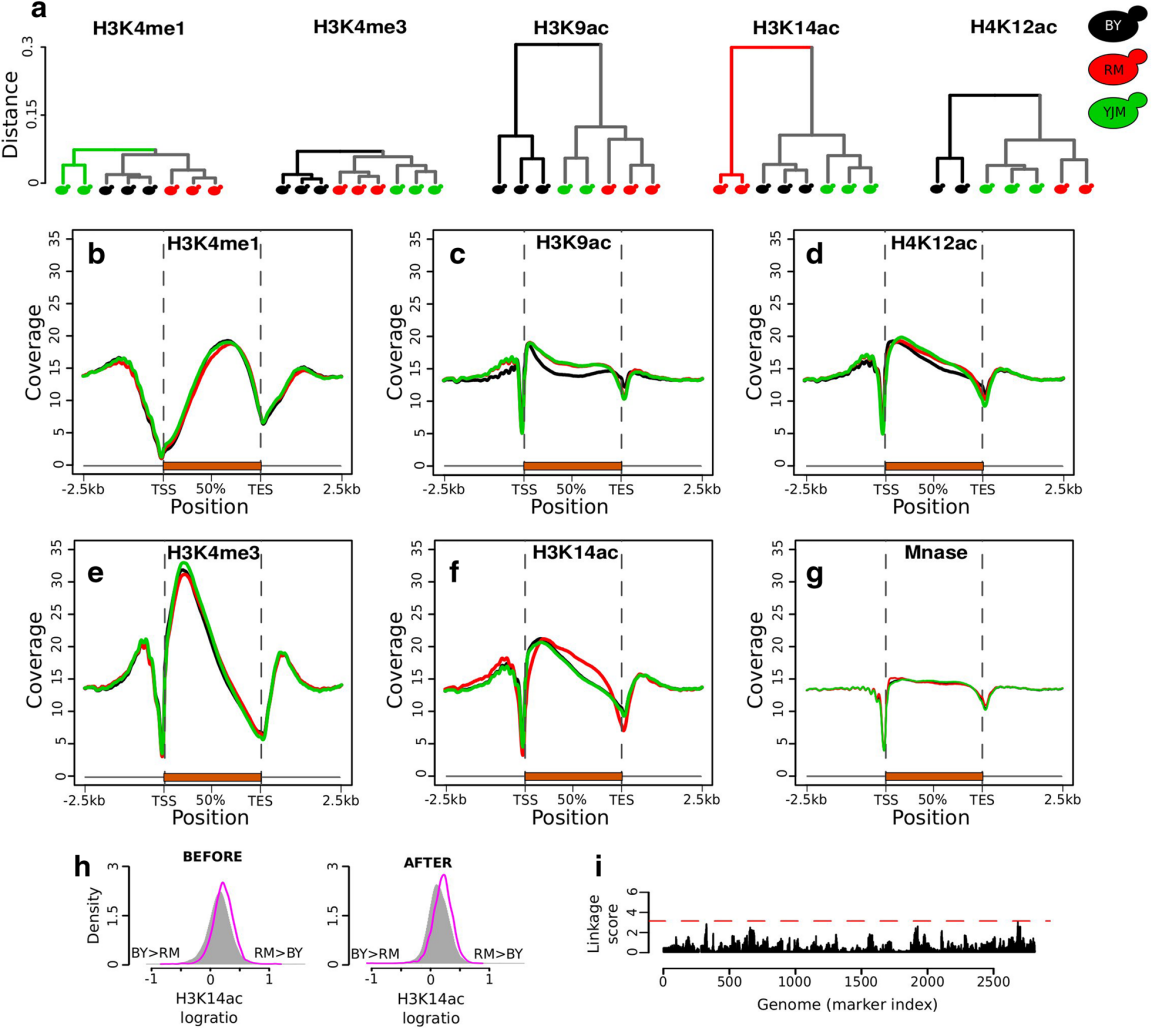
Inter-strain distances according to five epigenomic marks. **a** Hierarchical clustering of strains. For each histone modification, the distance between two samples was determined as *1* − *ρ*, where *ρ* is the Spearman rank-based correlation coefficient between the profiles of the two samples. Profiles comprised ChIP counts computed at every nucleosome by *NucleoMiner2.0* [44] (see “Methods”). **b**–**f** ChIP coverage profiles of the indicated marks along an average gene (in per-million reads, normalized and averaged across replicates). *Colors* correspond to strains as in **a**. **g** Same representation but for MNase average profile. **h** H3K14 acetylation differences between the BY and RM strains, before and after the transient reprogramming described in [15]. The distribution of log2(RM/BY) of ChIP-CHIP intensities is shown for all nucleosomes (*gray*) and for the subset of nucleosomes (*magenta*) located in the second half of the body of 529 genes responsible for the specific pattern of RM. *Magenta* and *gray* distributions significantly differ (Kolmogorov–Smirnov *p* value <10^−15^) both before and after reprogramming. **i** Genomic QTL scan for regulators of the RM-specific K14ac profile in **f**. *Red line* significance threshold determined by permutations. Linkage score: −log_10_(*P*), where *P* is the nominal QTL *p* value.

We then examined the specific extent of inter-strain variation for each mark. To do this, we computed dissimilarities between samples based on the correlation between their epigenomic profiles of the mark and applied hierarchical clustering on these dissimilarities. The resulting trees revealed interesting properties of epigenomic variation among the strains (Fig. 2a). First, the three acetylation marks displayed greater variation than the two methylation marks (higher branches of the tree). This could simply be due to the fact that acetylation marks probe more nucleosomes. If strains differ only a little for each nucleosome, then the cumulative effect on the genome is greater for marks that are widely distributed. Another explanation would be that the two biochemical mechanisms vary to different degrees. For example, if strains differ in the concentration or dynamics of acetyl-CoA but not of *S*-adenosyl methionine (SAM) biosynthesis, then protein acetylation patterns may globally differ more between the strains than methylation patterns. However, the metabolomic data previously collected on BY and RM did report differential steady-state levels of SAM between the strains [25]. Acetyl-coA concentration also seemed to differ but to a lower extent (Sean Hackett and Josh Rabinowitz, personal communication). Thus, the higher divergence in acetylation than methylation cannot be directly attributed to pronounced differential levels of the donor group. It remains possible that the dynamics of acetyl-CoA availability may be critical, as highlighted by the very rapid changes in chromatin acetylation, but not methylation upon cell cycle re-entry from starvation [26]. In this regard, it is interesting to note that the concentrations of compounds located upstream (e.g., fructose-6-phosphate) and downstream (e.g., citrate) acetyl-coA along glycolysis were linked to the *ira2* locus [25], which coincides with one of the QTLs controlling the acetylation level of multiple nucleosomes [15].

Secondly, the trees revealed interesting epigenomic specificities of the strains. BY differed from the other two strains in H3K9ac, H4K12ac and H3K4me3, whereas RM differed in H3K14ac and YJM differed mildly in H3K4me1. This indicates that intra-species epigenomic diversity is modular, with some histone marks differing globally in some strains/individuals, and other marks differing globally in others. Compared with previous studies, it is surprising that we observed this modularity at the full genomic scale. In human lymphoblasts, about 6% of genomic regions analyzed by Kasowski et al. displayed variation in H3K27ac, which matched population ancestry groups, and equivalent matching was observed for H3K4me1 and H3K27me3 [8]. Therefore, we expected that unrelated yeast strains would display modular variation at given regions, but we did not anticipate observing pronounced deviations at the scale of the entire genome.

Finally, trees were radically different between H3K9ac and H3K14ac variation. For H3K9 acetylation, the BY strain differed from both RM and YJM, whereas for H3K14 acetylation RM was clearly distant from BY and YJM. This was unexpected, because H3K9 and H3K14 are both acetylated by Gcn5 [27, 28], and their acetylation profiles on the genome were described as very similar, with a common enrichment in highly transcribed genes [16, 18]. It is therefore surprising to see that a strain can display a specific H3K9ac, but not H3K14ac profile, and vice versa. This could result from differential targeting or activity of other lysine acetyl-transferases or deacetylases. For example, Sas3 can substitute Gcn5 in the acetylation of H3K14, but not of H3K9 [28, 29]. Similarly, deacetylation by Hda1 was reported for H3K9 [30], but not for H3K14.

We therefore searched for variants among BY, RM and YJM protein sequences of these and other histone modifiers that could explain strain specificities. Non-synonymous SNPs and/or short indels were present in 16 of the 23 proteins considered (Additional file 5). The most remarkable mutation was a premature stop codon in YJM that removed the C-terminal bromodomain of Gcn5. By binding to acetyl lysines, this domain is responsible for the cooperative and site-specific acetylation of nucleosomes by SAGA [31, 32]. It is surprising to see that, although the strain lacking this functionally important domain is YJM, BY and RM display distinguishable acetylation profiles of Gcn5 substrates H3K9 and H3K14. It is possible that (1) the mutation affects the dynamics rather than the steady-state levels of acetylation, (2) that it affects only few genes or (3) that mutations in other genes compensate for it. Unlike Gcn5, Sas3 contained mutations in BY that might explain the particular H3K9ac profile in this strain. In addition, several of these genes displayed differential expression between strains. Direct manipulation of these genes is needed to determine their contribution to chromatin divergence, especially since our previous genome-by-epigenome linkage analysis showed that divergence arose from multiple other genetic sources [15].

Where are these global epigenomic specificities located relative to protein-coding genes? At the genomic scale, we computed averaged ChIP-seq profiles over all genes and visualized them for each strain (Fig. 2b–g; Additional file 6). These profiles were consistent with previous observations [16, 18] and showed interesting strain specificities. Strain BY displayed reduced acetylation of H3K9 in the middle of genes and of H4K12 in the second half of genes. Strain RM showed a redistribution of H3K14 acetylation compared with the other two strains, with a higher level in promoters, a lower level immediately downstream the transcription start site, a higher level in the second half of the gene body and a lower level around the transcription end site (TES). As mentioned above, the fact that both H3K9 and H3K14 acetylation showed a specific, but different pattern, was unexpected.

The redistribution of H3K14 acetylation in RM confirmed our previous comparative analysis by ChIP-CHIP [6]. The fact that YJM showed a similar profile to BY indicated that specific regulation of H3K14ac occurred in the RM strain. What these regulations are is unclear; nonetheless, we made two observations based on previous data. In a previous study, we quantified the persistence of BY/RM H3K14 acetylation differences over a reprogramming experiment. This was done by culturing the strains first in the presence of a high dose of trichostatin-A, an inhibitor of HDACs, and then in normal conditions for a prolonged time. Comparing again BY and RM H3K14 acetylation after this treatment and recovery allowed to determine which of the inter-strain differences were particularly labile or persistent [15]. Here, we re-analyzed this dataset to determine if the RM-specific profile of H3K14ac had persisted. We selected 529 genes having a pronounced BY/RM difference in H3K14ac ChIP-seq profile in the present study and extracted the nucleosomes located in the last two-thirds of the bodies of these genes. We then extracted from the previous dataset the log(RM/BY) ChIP-CHIP intensities at these nucleosomes, both before and after the transient perturbation (Fig. 2h). This showed that the high H3K14 acetylation of these nucleosomes in the RM strain persisted after recovery from the epidrug treatment. The specific regulations of H3K14ac taking place in the RM strain are therefore robust to such transient perturbations. This robustness could result from genetic control. If DNA polymorphisms of the RM strain regulate the redistribution of H3K14ac, then the specific acetylation profile would be re-established after perturbation. We previously described the genetic control of H3K14ac BY/RM epigenomic variation and found no evidence of such master regulators [15]. However, the statistical power of that analysis was limited by the multiplicity of traits considered in the study and it remained possible that regulators of a single and global trait had been missed. Thus, we re-analyzed this genetic data and specifically searched for genetic loci having a global effect on the acetylation profiles along gene bodies. We did this by constructing one metaphenotype that quantifies, in each BY × RM segregant, the degree of imbalance of H3K14ac in the body of 529 relevant genes. No statistically significant locus was detected by this dedicated genome scan (Fig. 2i), which confirmed that the global redistribution of H3K14ac in RM is not caused by one or few master genetic regulators. If the origin of the RM-specific genic profile of H3K14ac is genetic, it is likely driven by multiple small effect loci.

### Yeast genes differ in their pattern of natural epigenomic divergence

**Fig. 3:**
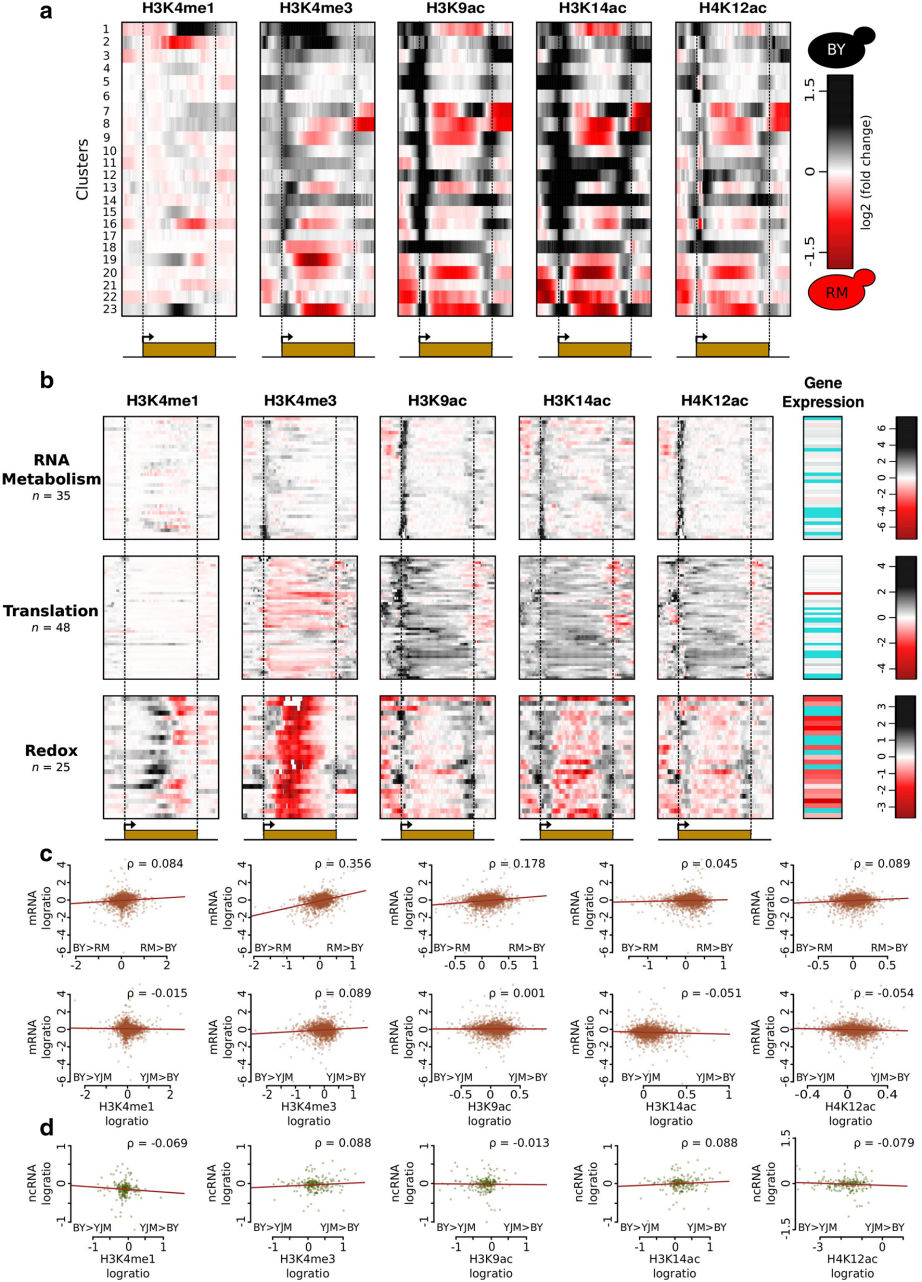
Gene clustering according to BY–RM epigenomic divergence. For each gene, the ChIP/MNase log ratio in each strain was computed on genomic bins covering the gene body (from TSS to TES, *orange box*, divided in percentiles) together with 500 bp of their upstream and downstream regions. The difference in signal between the two strains was computed (*black* to *red color scale*). Genes were clustered by their similarity in this differential signal across the five chromatin marks. **a** Average pattern of the 23 clusters described in Table 1. **b** Details of three clusters with previously measured differential mRNA expression [33]. *Cyan* missing mRNA data. **c** Correlation between gene expression (y-axis) and histone modification (x-axis) variation. For each modification, inter-strain difference was computed as the mean of (log_2_(ChIP/Mnase) of strain 1 − log_2_(ChIP/Mnase) strain 2) from the TSS to the TES. Each *plot* corresponds to one histone modification compared between two strains, with *dots* representing genes. Only genes for which the mean log ratio was above zero in at least one strain were considered. *Upper panels* BY versus RM. *Lower panels* BY versus YJM. *ρ* Pearson correlation coefficient. *Lines* linear fits. **d** Same analysis as in **c** but for the expression of non-coding transcripts in BY and YJM [23].

We then searched for shared or distinct patterns of variation among genes. We performed hierarchical clustering of the genes according to their differential profile of histone modifications between two strains. Importantly, differences in gene size did not seem to bias the epigenomic distances used for clustering, since no correlation was seen between the two (Additional file 7). Based on visual inspection of the entire clustering tree related to BY/RM differences, we extracted 23 gene clusters showing specific patterns of divergence between the two strains (Fig. 3a). Many clusters corresponded to elevated H3K4me3, H3K9ac and H3K14ac near the TSS in BY, which could result from higher expression of Gcn5, Sas3 and Set1 in this strain (Additional file 5). The genic patterns revealed two remarkable features. First, some clusters showed consistent co-variation of different histone marks, whereas others showed independent or negatively correlated variation of the different marks. For example, all four marks generally associated with active transcription (H3K4me3, H3K9ac, H3K14ac and H4K12ac) co-varied in clusters 9, 14, 20 and 23, but not in several other clusters. Genes of cluster 1 displayed lower levels of H3K4me3, but higher levels of H3K9ac and H3K14ac, in RM than in BY. Conversely, cluster 18 corresponded to higher levels of H3K4me3, but lower levels of H3K9ac, H3K14ac and H4K12ac, in RM than in BY. Cluster 2 grouped genes with increased mono-methylation and reduced tri-methylation of H3K4 in their bodies, with no remarkable variation of acetylation marks. When averaging all genes, co-variation of acetylation marks was also apparent in the BY/RM comparison, but not in the BY/YJM and RM/YJM comparisons (Additional file 8). The second observation was that variation could be restricted to particular portions of genes or was spread over the entire gene length. For example, the differential levels of H3K4me3, H3K9ac, H3K14ac and H4K12ac in genes of clusters 6 and 17 were focused at, or immediately downstream of, the TSS. Similarly, genes of cluster 4 varied in H3K9ac and H3K14ac specifically at promoter regions, and genes of clusters 3 and 10 differed in H3K9ac and H3K14ac at both TSSs and TESs, but not in between. In contrast, variation spread the entire length of genes from clusters 1, 2, 7, 8, 9, 14, 18, 20, 22 and 23. These results illustrated the complexity of natural epigenomic variation, with incomplete correlation between chromatin marks and localization within genes, which could be either focused or widespread.

Genes sharing similar patterns of variation may be functionally related. To examine this possibility, we searched every cluster for enrichment in gene ontology terms among the annotations of its genes. As indicated in Table 1, five clusters showed significant enrichments relating to amino acid catabolism, transmembrane transport, RNA metabolism, RNA translation (these genes are transcribed at high rate, Additional file 9) and oxido-reduction, respectively.

**Table 1:** Clusters of genes with similar patterns of epigenomic differences between the BY and RM strains

Cluster ID	Number of genes	GO term enrichment	BY versus. RM mRNA diff.
1	15	Serine family amino acid catabolic process	BY > RM
2	6	Transmembrane transport	–
3	52	None	BY > RM
4	32	None	BY > RM
5	85	None	–
6	12	None	–
7	40	None	–
8	31	None	RM > BY
9	477	None	RM > BY
10	1,389	None	–
11	56	None	RM > BY
12	22	None	–
13	29	None	RM > BY
14	40	None	–
15	141	None	–
16	195	None	–
17	35	RNA metabolic process	–
18	48	Translation process	–
19	25	Oxidation–reduction process	RM > BY
20	80	None	RM > BY
21	21	None	–
22	22	None	RM > BY
23	192	None	RM > BY

Given the known participation of histone modifications in transcriptional regulations, we examined if genes displaying chromatin divergence also displayed inter-strain differences in mRNA levels. The yeast strains used here have previously been an experimental model for comparative transcriptomics. Large datasets are available that quantify mRNA divergence in several environmental conditions, including standard growth conditions similar to the ones we used here. This allowed us to directly compare chromatin divergence patterns with the differential mRNA levels previously reported between the BY and RM strains [33] and between the BY and YJM strains [23]. As indicated in Table 1, of the 23 gene clusters corresponding to shared patterns of BY/RM chromatin differences, 11 showed evidence of transcriptional changes. One of them contained 25 genes sharing functional annotations related to oxido-reduction. These genes displayed high H3K4 tri-methylation in their gene bodies, accompanied by an elevated transcription in the RM strain (Fig. 3b, bottom). Interestingly, these 25 genes also showed this pattern of chromatin and transcriptional variation between the BY and YJM strains (Additional file 10, bottom), which argued for a specific down-regulation of both transcription and H3K4 tri-methylation of these genes in the BY strain. The remaining 12 clusters defined by BY/RM chromatin differences showed no such association with transcriptional changes (Table 1). In particular, the 35 genes of cluster 17, having a focused increase of acetylation in BY immediately downstream the TSS, were not particularly more expressed in this strain, which was also true when comparing BY with YJM (Additional file 10, top). Similarly, only one of the 48 genes of cluster 18, with contrasted tri-methylation and acetylation patterns between BY and RM, also varied in mRNA abundance (Fig. 3b). Such a partial co-variation of histone marks and mRNA levels was also observed when clustering genes on the basis of their epigenomic differences between the BY and YJM strains. In this case, 43 clusters could be extracted and evidence of gene expression variation was identified for 11 of them (Additional file 11).

Observing numerous genes where histone modifications and transcripts co-vary was expected. Several studies showed that eQTLs and histone modification QTLs tend to overlap [8, 9, 13, 15] and, in humans, histone mark variation could partially be attributed to the disruption of transcription factor binding sites [7–9].

We further examined the correlation between mRNA differences and variation of each of the five marks individually. As shown in Fig. 3c, H3K4me3 was the only mark to display co-variation with transcript levels in the BY/RM comparison. This is consistent with a recent study comparing the BY strain to an *S. paradoxus* strain, where variation of H3K4me3, but not of H3K9ac, was associated with transcriptional differences [34]. However, this was not true when comparing BY with YJM (Fig. 3c), indicating that generalization is not straightforward. Finally, differences in histone modifications between BY and YJM were also not associated with different levels of non-coding RNA (Fig. 3d).

Overall, our gene-centered analysis of histone mark variation showed that (1) groups of genes can share similar patterns of variation without significant mRNA changes and (2) histone marks mostly vary independently of expression changes except for H3K4me3 in one pair of strains. How can this be interpreted? Both chromatin and gene expression regulations are under complex regulatory control. The transcriptional activity of a gene is modulated by different types of signals, some related to the deposition of active histone marks and others related to transcription factors. These signals can be synergistic or antagonistic; they interact with one another, and each signal can have its own source of variation. The possibility that this complexity blurs the correlation between chromatin and transcriptional variation is supported by earlier observations. We and others showed that the genetic control of chromatin variation does not fully overlap with the control of gene expression. At least 30% of yeast QTLs of H3K14ac are not eQTLs [15], and 36% of the histone mark variation among a population of rats could not be attributed to variation in gene expression [13]. In addition, the genetic control of one histone mark sometimes compensates for the genetic control of another one. Among the QTLs controlling the antagonistic marks H3K4me3 and H3K27me3 at the same genes in rat tissues, 24% upregulated both marks, contributing compensatory actions on gene activity [13]. Similar complex scenarios are probably present in humans, because variability at multiple enhancers is needed to predict variable gene expression [8]. The correlation between chromatin and mRNA variation seems more detectable from heterozygous individuals displaying allelic imbalance [7]. This could be because allelic imbalance is not sensitive to the action of *trans*-acting factors that can antagonize *cis*-regulations in inter-individual studies. An example of *cis/trans* antagonistic control of H3K14ac levels was previously reported in BY × RM yeast strains [15]. Finally, we used transcriptomic data corresponding to steady-state mRNA levels, which, unlike RNA PolII ChIP, NET-seq [35] or DTA [36] do not reflect transcriptional activity itself. Turnover and post-transcriptional regulations of mRNAs probably also depend on various sources of variation, further attenuating the correlation between chromatin and transcript levels differences.

### Highly responsive and TATA box-containing genes show elevated epigenomic divergence

**Fig. 4:**
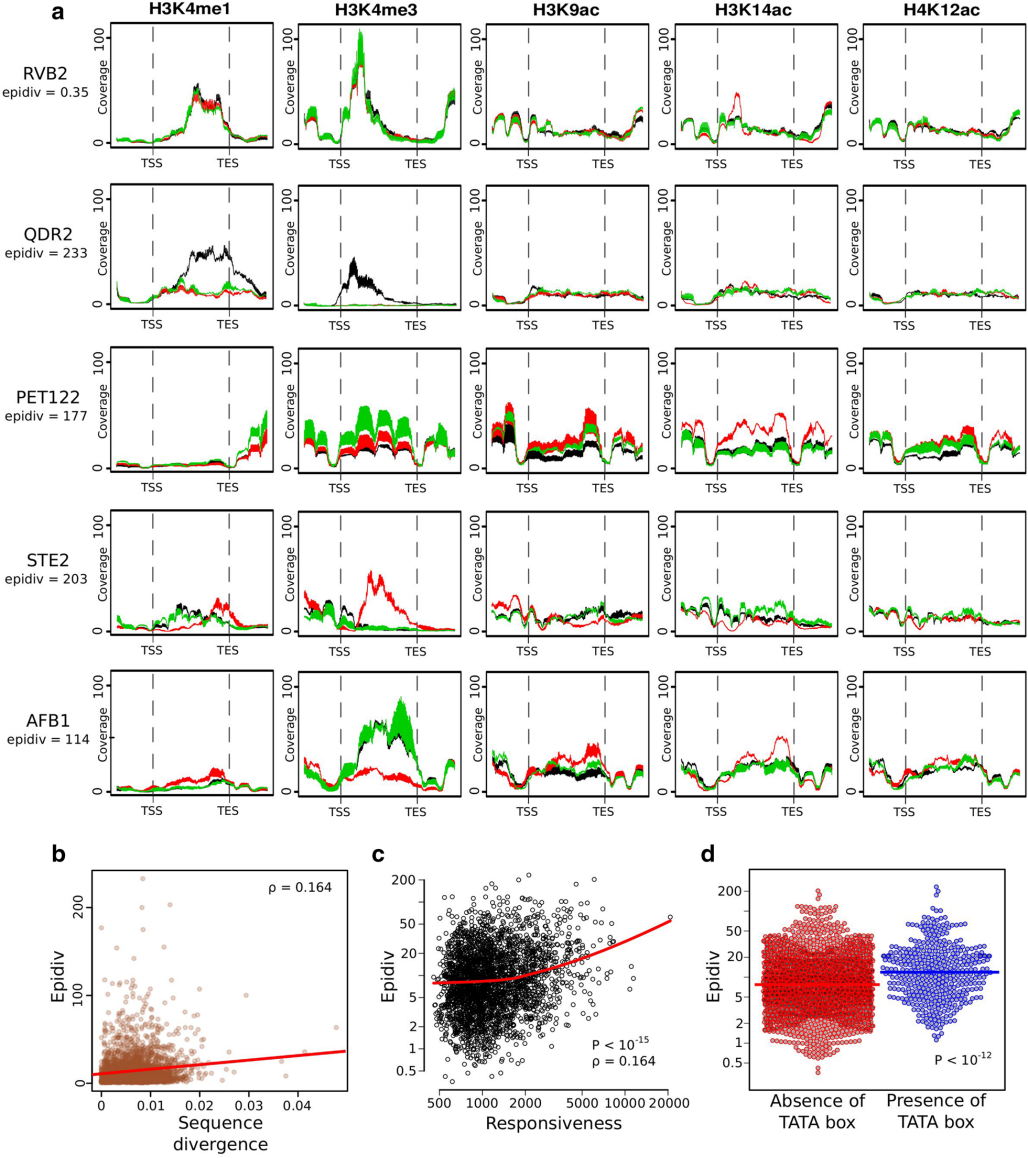
Intra-species chromatin divergence of every gene. Genes were segmented in bins corresponding to percentiles of the gene body (from TSS to TES) plus 500 bp of the upstream and downstream regions. For every gene, the divergence was quantified from an ANOVA model and termed ‘epidiv’ (see “Methods”). **a** Examples of genes with low (0.35) and high (>100) epidiv values. The normalized ChIP/MNase profiles are *colored* according to strains. *Black* BY, *red* RM and *green* YJM. **b** Epidiv values as a function of DNA sequence divergence of every gene. **c** Epidiv values as a function of transcription responsiveness from [60]. The *red line* is a smoothed average, showing that the correlation is mainly supported by highly responsive genes. *ρ* Spearman correlation coefficient. *P* value: significance rank-based correlation test. **d** Epidiv values for genes with or without a TATA box [60]. *P* value: Wilcoxon Mann–Whitney test. *Colored bar* median.

We asked if genes with the greatest intra-species epigenomic variation were associated with specific features. We computed a statistic (called ‘*epidiv*’ hereafter) that quantifies the extent of chromatin variation at every gene. This value corresponds to an interaction term in a linear model representing a strain-specific difference in at least one histone mark along the body of the gene (see “Methods”). Marked differences in *epidiv* values were observed between genes (Additional file 12). For example, *RVB2*, which encodes an ATP-dependent DNA helicase of the INO80 complex [37], displayed high conservation of epigenomic profiles with only one nucleosome having increased H3K14ac in the RM strain and decreased H3K9ac in the BY strain. The profiles of this gene and of genes with extreme *epidiv* values are shown in Fig. 4a. An example of medium *epidiv* value (*ISY1* gene) is shown in Additional file 13. Gene *QDR2*, which encodes a membrane transporter involved in multi-drug resistance [38], displayed variation in spanning from TSS to TES, with a remarkable enrichment of H3K4 mono- and tri-methylation in the BY strain, but little variation of other marks. The profiles observed were similar to those obtained when segmenting in absolute distance from TSS to TES (Additional file 13). A radically different pattern of elevated variation was seen for *PET122*, which encodes a mitochondrial translational activator. In this case, all five marks showed variation but with distinct patterns: YJM had increased levels of H3K4me1 downstream of the TES and of H3K4me3 on all four nucleosomes of the gene body; RM had high levels of H3K14ac along the entire gene; and BY had reduced levels of H3K9ac and H4K12ac. Other remarkable examples were the antagonistic variation of H3K9ac and H3K4me3 in the *LCL2* gene, and the specific and pronounced variation of H3K9ac in the *ATG17* gene (Additional file 13). Interestingly, the mating-type-specific genes *STE2,* expressed in RM (MATa), and *AFB1,* expressed in BY and YJM (MATalpha), also showed complex patterns, with high levels of H3K4me3 but not of other active marks in the expressing strains, a redistribution of H3K4me1 and an unexpected increase of H3K14ac in some non-expressing strains (Fig. 4a). A complex and more precise redistribution of marks was also apparent on some nucleosomes of the alpha-specific *SAG1* gene (Additional file 13). Finally, gene *NIT1*, encoding a nitrilase [39], showed another striking case of epigenomic divergence. Strain BY, where expression of this gene was higher than in RM [33], displayed increased H3K4me3 and a redistribution of H3K4me1 toward the 3′ end (Additional file 13). At the DNA sequence level, BY contained a frameshift mutation that was absent in RM and YJM. It is therefore possible that its specific epigenomic pattern results from a feedback-regulated compensation of the protein activity.

Using gene-level *epidiv* values, we searched for genomic features associated with elevated epigenomic divergence. We first determined whether genes with high DNA sequence variation among the strains also displayed high epigenomic divergence. This was only partially true: when plotting *epidiv* values as a function of SNP and indel density, a weak positive correlation was observed (Fig. 4b). Observing such a correlation is consistent with the numerous *cis*-acting polymorphisms previously reported to control histone modifications [7–9, 13, 15]. The weak association shows that high epigenomic divergence could be observed in genes with conserved DNA sequences. In addition, we observed no correlation between *epidiv* values and the ratio of nonsynonymous versus synonymous sequence divergence (*dN/dS*; Spearman *ρ* = 0.02, using the adjusted values of Wall et al. [40]). Epigenomic signatures are, therefore, not particularly constrained in genes that are under negative selection at the protein level.

We previously found increased variability of H3K14 acetylation in genes with high expression evolvability [6], and a similar observation was reported recently for H3K4me3 [34]. To examine this property further, we tested for correlations between inter-strain epigenomic divergence and various measures associated with gene expression evolution. *Epidiv* showed a weak positive correlation with expression mutational variance estimated from mutation accumulation lines (Wilcoxon *P* < 10^−7^ when testing the difference in *epidiv* between *‘*evolvable’ and ‘non-evolvable’ genes defined in [41]), with inter-species expression divergence [42] (Spearman *ρ* = 0.14, *P* < 10^−11^) and with transcriptional responsiveness to various environmental or genetic perturbations [42] (Spearman *ρ* = 0.164, *P* < 10^−15^), and it was slightly reduced in essential genes (Wilcoxon *P* = 0.002). Consistently, *epidiv* was also associated with strain-specific expression response to different growth conditions (Spearman *ρ* = 0.1, *P* = 10^−7^, between *epidiv* and the statistical significance of the strain-by-environment interaction term described in [33]). Importantly, these correlations were mainly supported by few genes with very high expression divergence or responsiveness (Fig. 4c). These genes were previously shown to frequently contain a TATA box [42]. Consistently, we observed a mild, but statistically significant difference in *epidiv* values between TATA-less and TATA box-containing genes (Fig. 4d). Note that this does not imply that TATA boxes themselves favor epigenomic diversity, as they may have co-evolved with other *cis*-acting determinants. We also noticed a slight increase in epigenomic divergence in genes transcribed at very low rates in standard conditions (Additional file 14). This is consistent with many highly responsive genes being poorly transcribed in these conditions [43]. Taken together, these results demonstrated a statistical association between epigenomic variability and transcriptional evolvability.

Given that half of the genomic bins used to compute *epidiv* are bigger for long genes, it is important to exclude that this association results from differences in gene size. Although small genes displayed higher *epidiv* values (Additional file 15), the association with TATA box remained when comparing genes of similar size (Additional file 16). We also re-calculated *epidiv* values using only fixed-size bins flanking the TSS (independent of gene size) and observed the same association with expression divergence (Spearman *ρ* = 0.11, *P* = 10^−6^), responsiveness (*ρ* = 0.24, *P* < 10^−15^) and TATA box (*P* < 10^−15^) (Additional file 17). Thus, this association is not a by-product of gene size differences.

What does this association imply? Chromatin diversity probably diversifies gene expression regulations. Genes with high *epidiv* values in our study may display inter-strain mRNA divergence under wild or stressful environmental conditions. It is also likely that the chromatin landscape modifies the regulatory effect of de novo mutations. For example, a mutation in a transcription factor may have negligible effects on the expression of target genes if these genes are in a repressive chromatin context. Diversity of chromatin states could therefore underlie diversity of fitness effects for regulatory mutations, increasing the evolutionary possibilities for gene expression. Further studies are needed to determine whether such chromatin-by-environment and chromatin-by-mutation interactions shape gene expression evolution.

### Extent of natural variation in nucleosome positioning

We next quantified inter-strain variation at every nucleosome individually. To do this, we developed a statistical framework, called *NucleoMiner2.0*, which aligned the genome of the strains, inferred nucleosome positions, determined the reproducibility of positioning across biological replicates, matched nucleosome maps of two different strains and tested for differential level of histone modification at every nucleosome. This data-processing pipeline is free and open-source and its details will be presented elsewhere [44]. The algorithm defines two types of nucleosomal regions: well-positioned nucleosomes, which correspond to individual nucleosomes displaying reproducible positioning across biological replicates; and “fuzzy” nucleosomes, which correspond to regions occupied by one or more nucleosomes having variable positioning between replicate experiments. Note that this definition of fuzziness is slightly different from the one commonly used in single experiments (e.g., peak width/height ratio), but the two are highly connected. When comparing two strains, two types of regions are determined: matched well-positioned nucleosomes, which correspond to individual nucleosomes that are well positioned in both strains and whose positions are consistent between the strains; and unaligned nucleosomal regions (UNRs), which correspond to the remaining regions occupied by one or more nucleosomes in at least one strain.

**Fig. 5:**
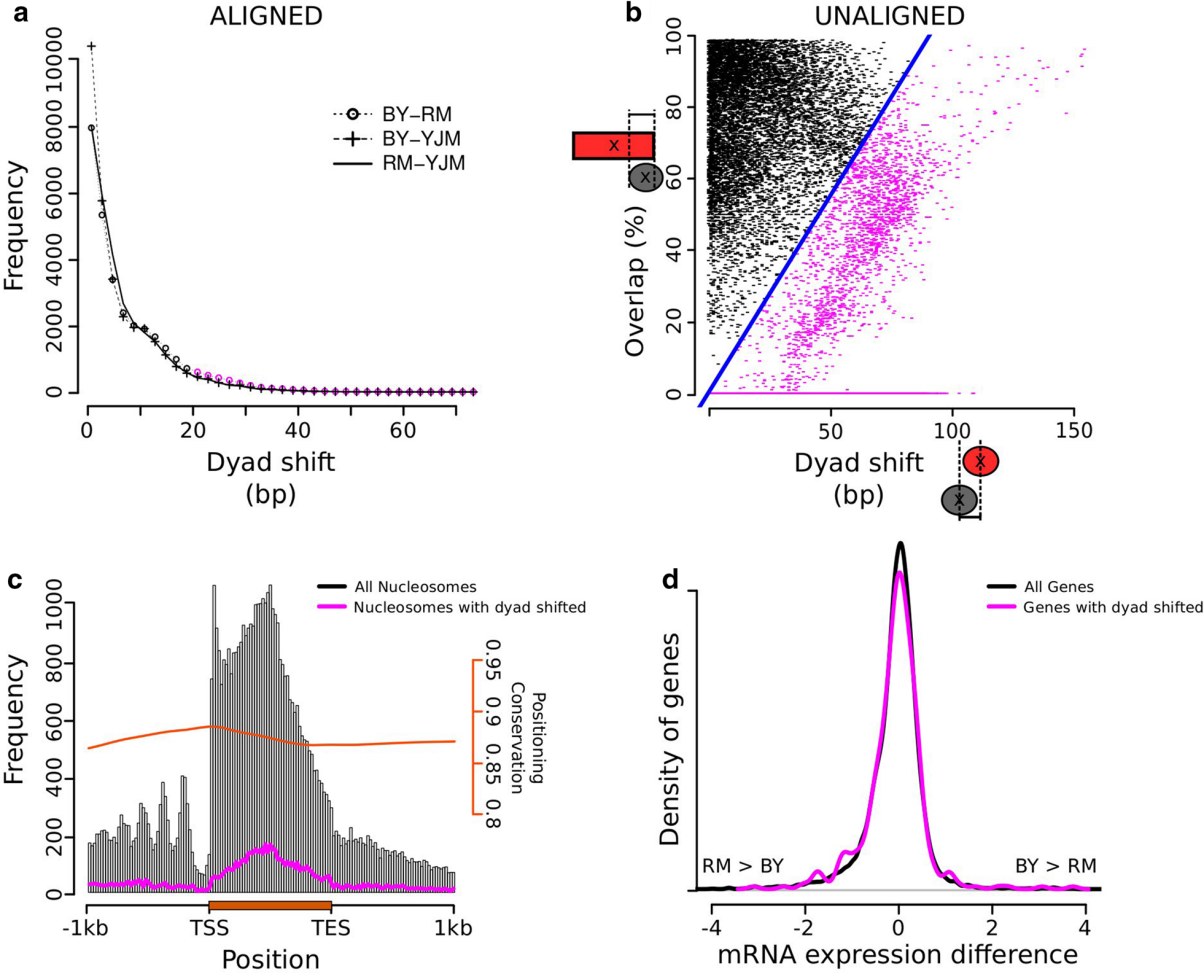
Divergence in nucleosome positioning. Our nucleosome mapping method defined two types of nucleosomal regions: well-positioned nucleosomes and UNRs, which correspond to conserved and variable positioning across biological replicates, respectively. **a** Shift in dyad position between strains for matched well-positioned nucleosomes. *Lines* show the distribution of the shifts between two strains. This* panel* considers the well-positioned nucleosomes of both strains that could be reliably matched. **b** Positioning divergence of nucleosomes that are well positioned in BY, but did not match a well-positioned nucleosome of RM. For all such BY nucleosomes (*gray ellipse*), two measures were retrieved that reflect distance to the nearest UNR (*red rectangle*) and nearest well-positioned nucleosome (*red ellipse*) in RM. Cases of full overlap (>99%) with a UNR from RM are not displayed because they correspond to conserved occupancy between the two strains. The *blue line* distinguishes a subpopulation of BY nucleosomes (*pink dots*) whose positions differ in RM [poorly matching a well-positioned nucleosome of RM (high x-axis value) or incomplete overlap with a nucleosomal region of RM (low y-axis value)]. **c** Average genic location of nucleosomes with differential positioning. The distributions show the location of well-positioned BY nucleosomes along an average gene. *Gray* all. *Pink* subset of nucleosomes whose positions differ in RM (*pink* flagged nucleosomes in **a**, combined with *pink* flagged nucleosomes in **b**). The fraction of nucleosomes that are not shifted (*orange smoothed line*) reflects conservation. **d** Transcriptional divergence between BY and RM. X-axis: log2 ratio of mRNA levels (data from [33]). Y-axis: density of genes. *Black* all genes. *Pink* 411 genes containing at least one nucleosome with differential positioning (from **c**). Shoulders in the *pink* distribution indicate matches between differential positioning and differential expression. The mode at zero shows that, for most of these genes, differential positioning is not accompanied by differential expression.

About 40,000 and 25,000 well-positioned and fuzzy nucleosomes were mapped in each strain, respectively (Additional file 18). Maps of these nucleosomes are provided in Additional file 19. After comparing these maps between strains, we examined the extent of variation in positioning by considering the nucleosomes that were reproducibly well positioned in one strain and querying their correspondence in another strain. Of 40,643 well-positioned nucleosomes of the BY strain, 30,464 matched a well-positioned nucleosome in RM and 31,689 matched a well-positioned nucleosome in YJM. To visualize the conservation in positioning between these matched well-positioned nucleosomes, we plotted the distribution of the difference in dyad position in the two strains. For most of them, the positions differed by less than ten nucleotides (Fig. 5a). However, some pairs showed large differences (up to 75 nucleotides), which could represent functionally relevant differential positioning. This was the case for 2,928 nucleosomes in the BY/RM comparison. We then considered the well-positioned nucleosomes of BY that did not reliably match a well-positioned nucleosome of RM. In many cases, the misalignment created a UNR. In other cases, another well-positioned nucleosome of BY better matched the nearest well-positioned nucleosome of the RM strain, creating a partially overlapping UNR [44]. To look for these cases of positioning divergence, we plotted the fraction of overlap with a UNR as a function of the distance to the nearest well-positioned nucleosome of RM (Fig. 5b). This revealed a subpopulation of 4,818 nucleosomes that were well positioned in BY and did not match a well-positioned or fuzzy nucleosome in RM. Altogether, we identified 7,746 nucleosomes whose positions diverged between the BY and RM strains. Very similar numbers were obtained when comparing BY with YJM or RM with YJM (Fig. 5a; Additional file 20).

To see where these ‘shifted’ nucleosomes localized relative to gene positions, we plotted their frequency along an average gene and compared it with the frequency of all nucleosomes. Positioning divergence was evenly distributed along genes, except immediately downstream of the TSS (Fig. 5c). This was explained by a higher positioning conservation of +1 nucleosomes (those located immediately downstream of a TSS): only 3% of +1 nucleosomes were listed as ‘shifted’ between BY and RM, whereas 12% of all other nucleosomes were shifted (*P* < 10^−15^, Chi square). Also, DNA sequence polymorphism was reduced at +1 positions (mean polymorphism among strains within ±30 bp of 0.28 versus 0.32% for all other nucleosomes, Wilcoxon *P* = 0.01). Finally, nucleosomes that were ‘shifted’ in RM as compared to BY corresponded to regions of higher sequence polymorphism (0.63 versus 0.32%, P < 10^−15^) and the same was true when considering nucleosomes ‘shifted’ in YJM as compared to BY (0.62%).

Nucleosome eviction has been associated with increased transcriptional activity [45]. We asked whether the ‘shifted’ nucleosomes were located proximal to transcription factor binding sites. Using the map of MacIsaac et al. [46], we observed that 0.18% of ‘shifted’ and 3.1% of ‘conserved’ nucleosomes were located within 30 bp of a binding site. This difference in frequency was marginally significant (*P* = 0.05, Chi square test). Thus, positioning tends to be more conserved in the proximity of transcription factor binding sites. We then looked at the BY/RM mRNA fold change for genes containing a ‘shifted’ nucleosome (Fig. 5d). Expression changes were slightly more pronounced among these genes than among all genes: the proportion of genes with >2-fold expression difference was 10% for this set as compared to 7% for all genes. Notably, many genes containing shifted nucleosomes were not particularly differently expressed between the strains. Thus, as for histone modification marks, inter-strain differences in nucleosome positioning is partially associated with differential mRNA levels.

### Single-nucleosome epi-polymorphisms (SNEPs) of five histone modifications

**Fig. 6:**
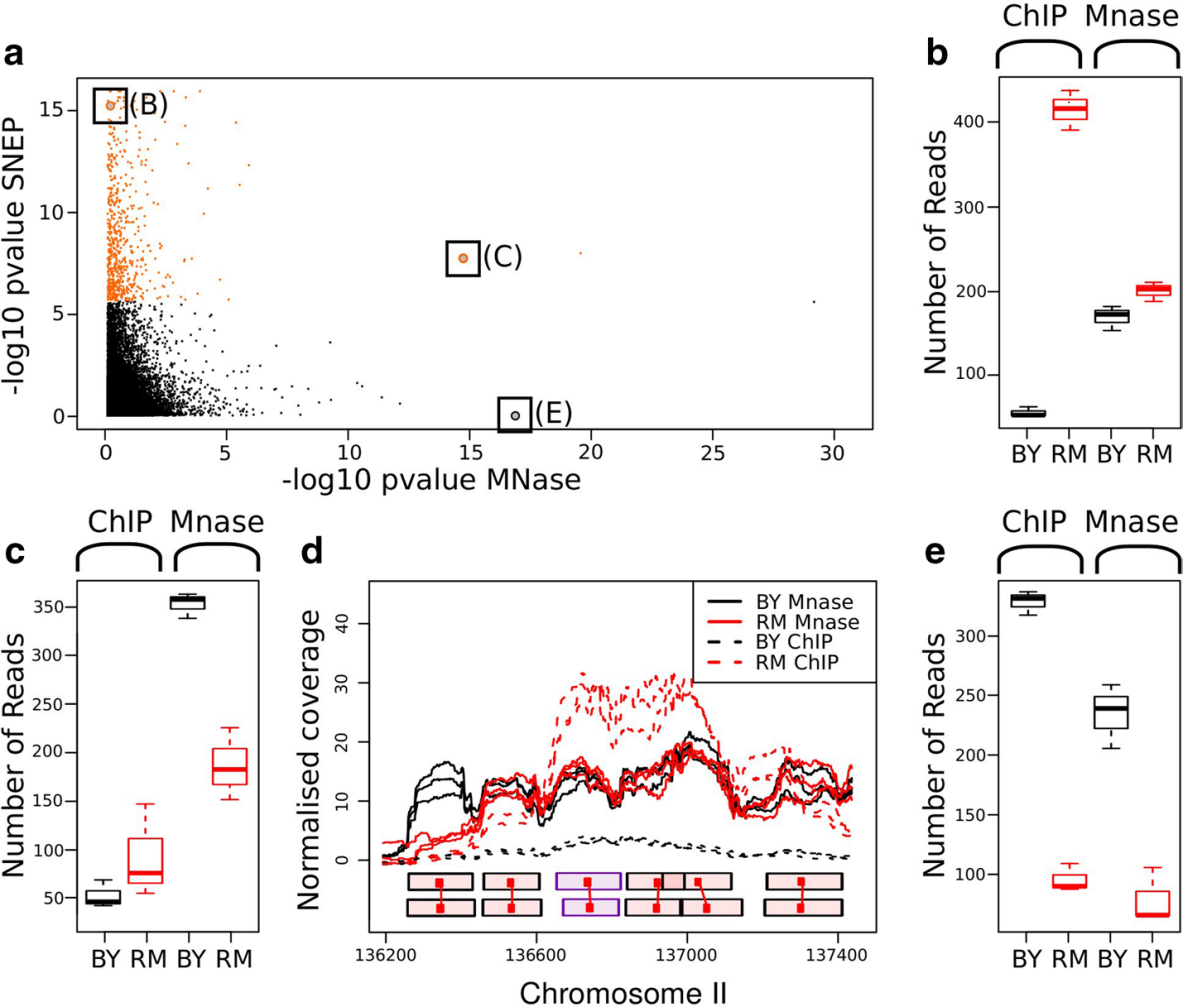
Statistical detection of SNEPs. **a** Statistical tests applied to 39,961 matched well-positioned nucleosomes and UNRs (*dots*). The x-axis is the significance of a differential MNase-seq signal between the strains, which reflects different levels of nucleosome occupancy. The y-axis is the statistical significance of SNEP detection for H3K4me3, which corresponds to the null hypothesis of no interaction term in a generalized linear model implemented in *DESeq* (see “Methods” and [44]). *Orange* (*black*) *dots* correspond to nucleosomes for which the test was (was not) significant at the genome-wide level, respectively (FDR = 0.0001). Labels *B*, *C* and *E* indicate nucleosomes presented in the corresponding* panels*. **b** Count data for a nucleosome where an SNEP is detected. A differential H3K4me3 ChIP-seq value was observed, with no significant change in MNase-seq counts between the strains. **c** Count data for a nucleosome where an SNEP is detected, with differential MNase-seq values. Despite a lower abundance of the nucleosome in RM, the ChIP signal for this strain is comparable or even higher than that for BY. The trimethylation level therefore differs between strains after accounting for nucleosome abundance. **d** Coverage profile of the locus containing the SNEP presented in **b**. The figure was produced by prolonging the reads to a final length of 150 nucleotides and normalizing by the sample size factor (see “Methods”). *Boxes* below the profiles indicate six well-positioned nucleosomes (*top* BY, *bottom* RM), colored in *violet* for the one presented in **b**. x-axis: genomic coordinates (in nucleotides) on the BY genome. **e** Count data for a nucleosome where the differential ChIP signal is fully explained by differential occupancy. Variation at this nucleosome is therefore not an SNEP.

We identified inter-strain variation on individual nucleosomes by considering differences in ChIP-seq counts while accounting for differences in MNase-seq counts (input). This corresponds to differential loading of the histone modification per occupied nucleosome, which meets the definition of an SNEP [6, 15]. As illustrated by the examples shown in Fig. 6 for H3K4me3, we detected multiple cases of differential occupancy of the nucleosome, numerous cases of differential ChIP counts and several instances where both ChIP and input counts varied between strains. On average, 3,150 SNEPs were detected at a false discovery rate (FDR) = 0.0001 in pairwise comparisons of the strains for the five histone modifications (Table 2a). This represented about 10% of the nucleosomes tested. Applying the test to UNRs revealed a similar proportion of regions with significant variation (Table 2b). This showed that the fraction of chromatin subjected to variation was large. Note, however, that the extent of variation (fold change) could be modest (Additional files 21, 22, 23). For all marks, the distribution of SNEPs relative to gene position was consistent with the average profiles of the marks shown in Fig. 2 (Additional file 24). Remarkably, five to ten times more SNEPs were found for H3K14ac than for the other modifications. This high proportion was driven by the RM strain and the numbers obtained were consistent with our previous estimates of H3K14ac variation [6], and with the divergence of the RM H3K14ac epigenome from the other strains (Fig. 2a). As mentioned above, the origin of this RM specificity is unclear.

**Table 2:** Number of regions with quantitative inter-strain differences in histone modifications (at FDR = 0.0001)

	Nucleosomes tested	H3K4me1	H3K4me3	H3K9ac	H3K14ac	H4K12ac
(a) Matched well-positioned nucleosomes carrying SNEPs						
BY–RM	30,464	478	744	73	3,549	20
BY–YJM	31,689	253	554	78	64	105
RM–YJM	30,421	48	257	24	3,186	10

	Regions tested	H3K4me1	H3K4me3	H3K9ac	H3K14ac	H4K12ac
(b) UNRs with significant differences						
BY–RM	9,497	302	257	107	1,559	46
BY–YJM	9,207	89	124	111	83	84
RM–YJM	9,935	32	60	29	1,248	10

**Fig. 7:**
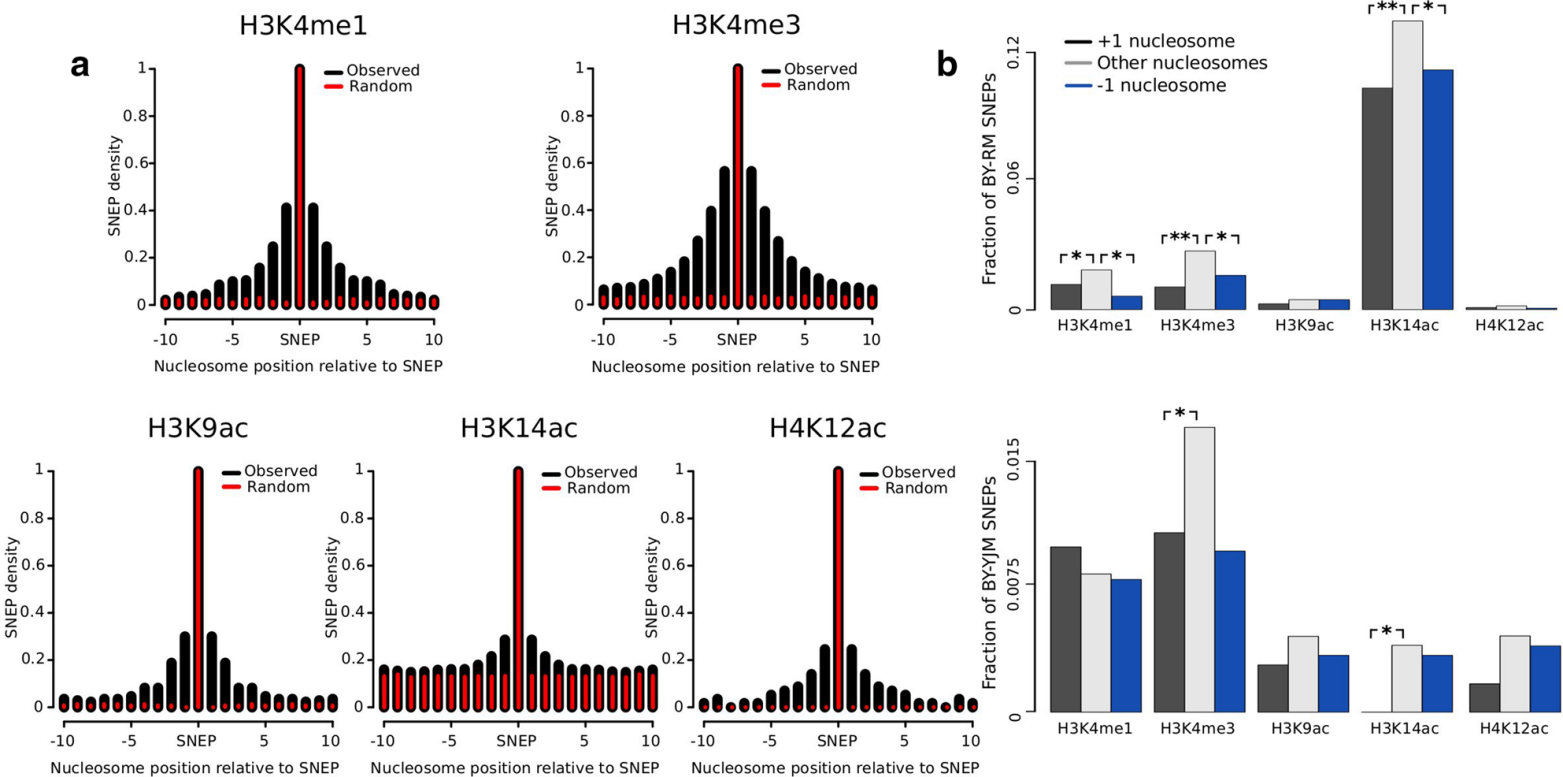
Regionality versus. precision of nucleosomal variation. **a** Analysis of BY/RM SNEPs. For each histone mark, regionality of variation was examined by counting, for each SNEP, the frequency of SNEPs on the ten upstream and ten downstream nucleosomes (*black bars*). Expected frequencies in the absence of regionality were estimated by re-assigning the SNEPs of this mark to random nucleosomes (*red bars*). Large *black* shoulders correspond to high regionality, where SNEPs tend to group together. Regionality of H3K4me3 remained when randomization was restricted to nucleosomes having above-background ChIP signal in at least one strain (Additional file 25, C). **b** SNEP frequency among +1, −1 and all other nucleosomes. Chi square test significance: **p value <10^−6^, *p value <0.01.

Genic patterns showed that variation could be focused at specific loci or spread over large regions (Fig. 3). To determine the proportion of SNEPs falling in loci of regional or focused variation, we plotted the fraction of nearby nucleosomes that were also SNEPs for the same histone mark (Fig. 7a). This showed a significant regionality of variation for all marks: nucleosomes close to an SNEP were more likely to carry similar SNEPs than random nucleosomes. We also observed different properties between marks: variation of H3K4 tri-methylation was predominantly regional, whereas H3K14ac variation was more focused (low shoulders in Fig. 7a; Additional file 25). Examples of regional methylation in the *QDR2* gene and focused acetylation variation in the *RVB2* gene are shown in Fig. 4a.

In summary, SNEPs were found on ~10% of analyzed nucleosomes; the majority of them corresponded to H3K14 acetylation variation; and variation in H3K4 tri-methylation tended to spread to consecutive nucleosomes, whereas variation in H4K12 acetylation was often focused on specific nucleosomes.

### Intra-species variation is reduced at +1 (and −1) nucleosomes

Nucleosomes located immediately downstream of a TSS are critically important in the regulation of transcription [47, 48]. Their positioning is strong and, as stated above, less variable between *S. cerevisiae* strains. Therefore, we asked if these nucleosomes also have reduced variability in histone modification levels. Compared with other nucleosomes, we observed fewer BY/RM SNEPs on these nucleosomes for all five marks and the difference was statistically significant for H3K14ac, H3K4me3 and H3K4me1 (Fig. 7b). Counting BY/YJM SNEPs showed the same trend for four of the five marks, although the total number of observations in this case was sometimes too low to reach statistical significance. Interestingly, nucleosomes located immediately upstream the TSS −1 position) also showed reduced variability (Fig. 7b). This contrasts with the known increase of genetic variants at these positions [49]. The BY/RM SNEPs that did occur for H3K4me3, H3K9ac, H4K12ac and H3K4me1 on +1 nucleosomes were associated with expression changes, but this was not the case for BY/YJM SNEPs (Additional file 26). Given their critical role in gene expression regulation, it is possible that the histone modification signature of these nucleosomes is under negative selection, thereby explaining the observed reduced variability.

### Frequent co-variation of different histone modification marks

**Fig. 8:**
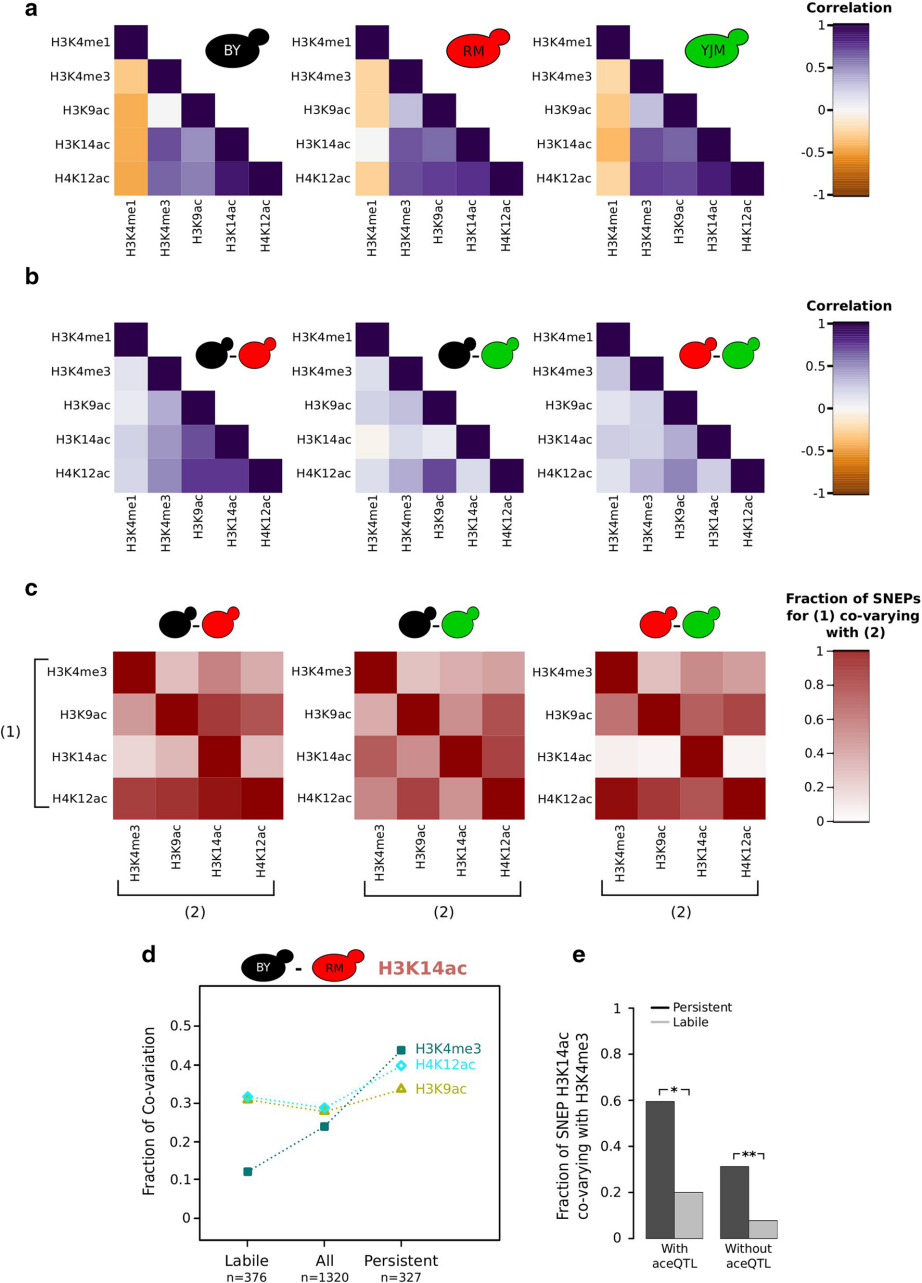
Co-variation of chromatin marks. **a** Correlation of epigenomic profiles in each strain. *Colors* represent Spearman coefficients of pairs of histone marks, computed on the genome-wide vectors of nucleosome-level ChIP/MNase signal. Low or negative correlations correspond to marks located on different nucleosomes. **b** Correlation between inter-strain difference in one histone mark and inter-strain difference in another mark. In each strain–strain comparison, the divergence of one mark was quantified on every nucleosome, while accounting for differential nucleosomal abundance (Fig. 6). *Color* Spearman correlation coefficient between such estimates of two histone marks, across all nucleosomes. **c** Fraction of SNEPs co-varying with another mark. For each strain pair, the set of nucleosomes harboring an SNEP of mark (*1*) was analyzed by counting how many showed significant divergence in mark (*2*) (at *p* value <0.01) in the same direction [higher level of mark (*2*) in the strain with higher level of mark (*1*)]. The *p* value corresponded to the nominal test used to detect SNEPs of mark (*2*). **d** Co-variation of specific H3K14ac SNEPs with other active marks. All ‘*labile*’ and ‘*persistent*’ SNEPs described in [15] were matched to nucleosomes of the current study and retained if matching was unambiguous and if H3K14ac SNEP significance in the current study verified *p* value <0.001. They were then analyzed by counting how many of them co-varied consistently with H3K9ac, H4K12ac, or H3K4me3 in the BY–RM comparison. **e** Co-variation with H3K4me3 is more frequent among persistent H3K14ac SNEPs than among labile H3K14ac SNEPs even when accounting for genetic control. The 327 persistent SNEPs (**d**) were split according to whether they were under the control of an aceQTL or not [15]. *Stars* indicate the significance of a Chi square test of independence at *p* = 0.012 (*) and *p* < 10^−13^ (**).

When a significant variation is observed for one mark on a nucleosome, is it accompanied by variation of another mark on the same nucleosome? We attempted to answer this question in three steps. First, we examined the extent of correlation (similarity of profiles) between marks within each strain. As expected from previous studies [16, 18], active marks were correlated and differed from H3K4me1 (Fig. 8a). Two interesting specificities were observed. The correlation between H3K9ac and H3K4me3 was weak in the BY strain. This is probably caused by gene promoter regions that have normal H3K4me3, but reduced H3K9ac in this strain (Fig. 2c). In addition, the negative correlation between H3K14ac and H3K4me1 was absent in the RM strain (Fig. 8a). This was consistent with the partial redistribution of H3K14ac in the downstream part of gene bodies, where H3K4me1 was maximal (Fig. 2f).

Second, we directly quantified the co-variation of distinct marks. Thanks to the scan for SNEPs described above using *NucleoMiner2.0,* the variation of each mark in each pair of strains was quantified for every nucleosome. Therefore, co-variation across all nucleosomes could be estimated by the correlation between the variation of one mark and that of another. We observed cases of substantial co-variation and different degrees of correlation between strain pairs (Fig. 8b). For example, the co-variation of all three acetylation marks was strong in the BY–RM comparison, weak in the RM–YJM comparison, and occurred only between H3K9ac and H4K12ac in the BY–YJM comparison. Notably, no mark co-varied with H3K14ac in the BY–YJM comparison when all nucleosomes were considered.

Co-variation of distinct active marks might be more pronounced on nucleosomes showing marked differences in at least one mark. We therefore focused on nucleosomes carrying SNEPs of active marks. For each mark, we counted the fraction of SNEPs that showed evidence of co-variation with another active mark. In all cases, this fraction was higher than the random expectation (Fisher’s exact test, *P* < 0.03), but the enrichment could be modest or pronounced (Fig. 8c). H4K12ac SNEPs consistently co-varied with the three other marks in all strain pairs. The co-variation of H3K14ac SNEPs with active marks was pronounced on comparing BY with YJM, weak on comparing BY with RM and nearly absent on comparing RM with YJM. Thus, the co-variation of H3K14ac with other acetylation marks on all nucleosomes in the BY–RM comparison (Fig. 8b) was not driven by nucleosomes carrying H3K14ac SNEPs. This also indicated that the majority of RM-related H3K14ac SNEPs were not accompanied by variation of other active marks, whereas the few H3K14ac SNEPs differing between BY and YJM were.

Finally, we asked if co-variation of active marks was more pronounced at sites showing regional variation (differential levels of the same mark on consecutive nucleosomes). To do this, we distinguished ‘regional’ BY/RM SNEPs, where one of the flanking nucleosomes also carried an SNEP for the same mark, from ‘isolated’ BY/RM SNEPs, where none of the flanking nucleosomes did. The fraction of SNEPs co-varying with another mark was similar in the two categories (Additional file 27), indicating that regionality of variation was not generally associated with variation of multiple marks.

### Persistent, but not labile H3K14ac SNEPs co-vary with H3K4me3

We showed previously that H3K14ac SNEPs could be partially reprogrammed after a transient treatment with trichostatin-A (TSA, an inhibitor of histone de-acetylases) followed by prolonged recovery. This earlier study was based on microarrays and identified SNEPs (termed ‘*labile*’) that were significantly affected by the treatment/recovery protocol and SNEPs (termed ‘*persistent*’) that remained different between the strains. The study also included an independent genetic mapping of DNA polymorphisms regulating H3K14ac levels (aceQTL), which revealed that, as expected, SNEPs under genetic control displayed higher persistence [15]. However, this association between genetic control and persistence was only partial, leaving the possibility that persistence of H3K14ac SNEPs could also result from non-genetic sources. In particular, if other active chromatin marks differed between BY and RM at the same loci, then these marks might contribute to the maintenance or re-establishment of H3K14 acetylation differences.

This possibility can, at least partly, be investigated here. If other active marks confer persistence to H3K14ac variation, then the nucleosomes containing persistent H3K14ac SNEPs should show variation in these marks. We therefore counted how many persistent and labile SNEPs showed evidence of synergistic variation in the level of H3K9ac, H4K12ac or H3K4me3. As shown in Fig. 8d, the fraction of H3K14ac SNEPs co-varying with H3K9ac was similar for persistent or labile H3K14ac SNEPs (~30%). The fraction co-varying with H4K12ac was slightly higher when analyzing persistent (40%), as compared to labile (31%) H3K14ac SNEPs. Strikingly, the fraction co-varying with H3K4me3 was several times higher among persistent (44%) than among labile (12%) H3K14ac SNEPs. This revealed that inter-strain differences in H3K4me3 levels correlated with the persistence of H3K14ac differences. However, this correlation might be causal (differences in H3K4me3 conferring persistence to H3K14ac differences) or not (another factor, such as a DNA polymorphism, controlling both marks in a persistent manner).

We therefore asked if the correlation resulted from a common genetic control of both H3K14ac and H3K4me3. Using the map of genetic modifiers of H3K14 acetylation (aceQTL), we distinguished the genetically controlled H3K14ac SNEPs from those for which no genetic control was found. The correlation between H3K14ac SNEP persistence and their co-variation with H3K4me3 was significant in both categories of H3K14ac SNEPs (Fig. 8e). Thus, even when no genetic control of H3K14ac variation was detected, its persistence could be associated with co-variation of H3K4me3. Although it remains possible that undetected genetic modifiers control both marks, this observation suggested that inter-strain differences in H3K4me3 confer persistence to H3K14ac SNEPs.

It is remarkable that H3K14ac SNEP persistence could be associated with co-variation of a methylation mark and not of acetylation marks. The transient perturbation of chromatin was achieved by TSA, which inhibits histone de-acetylases, but not de-methylases. It is therefore likely that differential levels of H3K4me3 and other methylation marks were not affected by the treatment. Persisting differential levels of histone methylation could then induce the re-establishment of differential levels of H3K14ac during recovery, via cross talk between the marks. Although still poorly understood, such cross talk exists. For example, deletion of the SET2 histone methyltransferase modifies the genic distribution of H3K14ac, which is associated with appearance of cryptic transcription [18]. Histone methylation (especially H3K4me2 and H3K36me3) could also increase the recruitment of NuA4, a lysine acetyl-transferase, which in turn facilitates the recruitment of SAGA, a major acetyl-transferase complex targeting H3K14 [50]. Conversely, histone hypo-acetylation could induce hypo-methylation via the recruitment of the Jhd2 demethylase [51]. Determining which cross talk mechanism underlies the contribution of H3K4me3 differences to H3K14ac SNEP persistence requires additional dedicated experiments.

In conclusion, we propose that a fraction of H3K14ac inter-strain differences can be robust to a transient TSA treatment via cross talk and synergy with differential levels of H3K4me3 and, perhaps, other methylation marks.

### What does quantitative variation imply?

Finally, we emphasize on the biological importance of observing quantitative, often subtle, differences between the strains. Quantitative differences imply that, in a cell population, only a fraction of the cells possess the histone modification at certain positions and that this fraction differs from one strain to another. How is the population affected by this fraction of cells? If the presence of active marks renders these cells more responsive to transcriptional activation, then a higher fraction of such cells can provide the population with a better potential of adaptation. However, if the response involves costly cellular decisions (e.g., arrest from cell cycle, synthesis of protective components or entry in meiosis), then triggering the response when it is not needed is counter-productive. In this case, a population with fewer cells carrying the active mark is better fit. It is possible that the different environmental history of the strains, especially the dynamics of the challenges they experienced, contributed to establish different fractions of cells carrying histone marks at certain positions.

## Conclusions

Understanding the complex pattern of epigenomic variation within a species is a key component of population epigenomics. In this report, we quantified this variation in yeast at individual nucleosomes using a new statistical framework. We generated maps of nucleosomes harboring inter-strain variation for H3K4me3, H3K9ac, H3K14ac, H4K12ac or H3K4me1. For every modification, variation was abundant and complex, with partial association with the variation of gene expression. Genes with evolvable or highly responsive expression displayed higher chromatin variability, and +1 nucleosomes were less variable than other nucleosomes. Co-variation between marks was detected and the data suggested that H3K4me3 differences could help re-establish H3K14ac differences after reprogramming. However, co-variation was not systematic, with surprisingly distinct patterns of variation between the functionally related H3K9ac and H3K14ac modifications. The complexity found in this pilot survey in yeast suggests that modularity and high complexity can be anticipated in population epigenomics of higher eukaryotes, including humans.

## Methods

### Strains

Yeast strains used were BY4716 *MATalpha lys2Δ0* [52], RM11-1a *MATa leu2Δ0 ura3Δ0 hoΔ::KanMX* [21] and YJM789 *MATalpha lys2 gal2 ho::hisG* strain [19].

### Single-nucleosome ChIP

Yeast cells were grown to exponential phase in flasks containing synthetic medium with 2% glucose (SDall) at 30°C. Cells were processed for MNase-seq and MN-ChIP-seq as previously described for MNase-CHIP and MN-ChIP-CHIP [6]. Briefly, cells were fixed with formaldehyde and their cell wall was digested with zymolyase. After lysis, samples were digested with micrococcal nuclease. Digestion was controlled on 2% agarose gel to verify the predominance of a single band at 150 bp. For MNase-seq, DNA was recovered and processed for library construction. For MN-ChIP-seq, immunoprecipitation was performed using: 10 µl anti-H3K4me1 polyclonal antibody (ActiveMotif, 39297), 3 µl anti-H3K4me3 polyclonal antibody (Diagenode, CS-003-100), 8 µl of anti-H3K9ac polyclonal antibody (Abcam, ab10812), 3 µl of anti-H3K14Ac polyclonal antibody (Upstate, 07–353) and 4 µl of anti-H4K12ac polyclonal antibody (Upstate, 07-595), respectively. All strain/antibody combinations were performed in three biological replicates (independent cultures).

### Library construction and sequencing

Illumina sequencing libraries were built using BioScientific NEXTflex™ ChIP-Seq Kit and NEXTflex™ ChIP-seq Barcodes, adapting the manual with the following modifications: we did not include a pre-size selection PCR and agarose gel size selection, and we applied only ten cycles of amplification. DNA library concentration was quantified using the Qubit fluorometer (Life Technologies) and its size distribution was verified on a Bioanalyzer system (Agilent). One library (H3K4me1, strain YJM) had very low concentration and was discarded. All other libraries were sequenced in single read mode, 50 bp long, on a Illumina HiSEQ 2000 sequencer at ViroScan3D/ProfileXpert (Lyon, France).

### Reads mapping

The genome sequences of S288c (isogenic to BY) and RM were downloaded in December 2007 from NCBI (ftp://ncbi.nih.gov/genomes/Saccharomyces_cerevisiae) and the Broad Institute (http://www.broad.mit.edu/annotation/genome/saccharomyces_cerevisiae/Home.html), respectively. The genome of YJM was downloaded from http://www-sequence.stanford.edu/yjm789_public/yjm-download.html in February 2009. Reads were aligned using *Bowtie 2* (2.0.0-beta7) [53], with option “–very-sensitive”. Alignments were collected in SAM format, converted into BAM files using *SAMTools* (0.1.18) [54] and then into BED files using *BEDtools* (v2.16.2) [55].

### Pairwise genome alignments

Genomes of two strains were aligned using *MUMmer* [56] and custom scripts developed in version 1.0 of *NucleoMiner* [6]. This step produced a.*c2c* file listing all similarities and polymorphisms. We used this file to define regions that are common to both genomes, larger than 4 Kb and containing no indel greater than 30 bp. These regions of interest were called common uninterrupted regions (CUR) [44]. All statistical analyses were then done using R (http://www.r-project.org).

### Coverage profiles

We built coverage profiles of every experiment as follows. Every CUR was segmented in 90 bp chunks. For each chunk, the coverage of one experiment was computed by translating the coordinates of the chunk in the genomic coordinates of the strain used in this experiment, and counting the number of forward reads having a start position within the chunk. Values were converted in per-million reads and divided by the chunk size to account for (1) shorter chunks truncated at the border of CURs and (2) chunks with length different from 90 bp after translation, because of the presence of indels. We then inspected the consistency of coverages across biological replicates by plotting the distributions of the values obtained. Triplicates displayed similar distributions except for one (H3K9ac, YJM) sample, one (H4K12ac, RM) sample, one (H4K12ac, BY) sample and one (H3K14ac, RM) sample, which markedly deviated from their corresponding replicates. The data of these four samples were discarded from further analysis.

### PCA analysis

For simplicity, only forward reads were used because reverse reads contained redundant information (dosage of the same nucleosome, at the other extremity). The matrix of coverages (columns = experiments, rows = chunks) was normalized by dividing each value by the sum of its column. PCA was performed using the *prcomp()* function in R. To assess the significance of the components, PCA was run three times on permuted datasets. On average, the fraction of variance explained by the first component on permuted data was 2.3%.

### Gene-level statistical analysis

IDs of genes in clusters are provided in Additional file 28. ChIP coverage profiles of all biological replicates along an average gene are shown in Additional file 29. Genes that were longer than 100 bp and which were fully included in CURs (3,078 genes in total) were segmented in 100 bins from TSS to TES, plus 250 bins of 10 bp upstream and downstream. For every bin, the per-million reads coverage of each sample was normalized using the size factor of the sample computed by *DESeq* [57], and replicates were averaged. For Fig. 2b–g, profiles were averaged across all genes. For gene clustering, profiles were transformed in ChIP/MNase log-ratios. Nine genes displaying near-zero signal for all marks were discarded. For the remaining 3,069 genes, the five profiles (one per histone mark) were truncated to keep only 500 bp upstream TSS and downstream TES, and were concatenated in a single vector (one vector per gene). The differential pattern between two strains was then obtained by subtracting the resulting vector of one strain from the vector of the other strain. Hierarchical clustering was then performed on these differential patterns, using complete linkage (Fig. 3a, b). For each gene, *epidiv* was computed from an analysis of variance (ANOVA) with the linear model:

$${\text{coverage}} \sim \, {\text{strain}} + {\text{mark}} + {\text{bin}} + {\text{strain}}{:}{\text{mark}} + {\text{strain}}{:}{\text{bin}} + {\text{strain}}{:}{\text{mark}}{:}{\text{bin}} + \varepsilon ,$$

where coverage is the ChIP/MNase log ratio mentioned above at genomic bin bin for histone mark mark in strain strain. Colons indicate interaction terms in the model and ε the residual error. The term of the model reflecting inter-strain divergence across the entire gene was strain:mark, and *epidiv* was defined as the F statistics associated with this term (Fig. 4). Note that (1) we did not use this term to reject the null hypothesis that it is zero, but to capture the amount of variation according to the model and (2) the term strain:mark:bin captures strain-specific redistribution of some marks along the gene, which is also relevant to divergence in chromatin pattern. However, this triple interaction term is equally affected by local variation between nearby bins as by more pronounced redistribution between distant bins. The biological interpretation of the proportion of variance explained by this term is not straightforward.

### QTL scan for determinants of RM-specific H3K14ac enrichment in 3′ of genes

As stated above, the chromatin profiles of each gene consisted of ChIP-seq signals computed on physical segments of the gene body. Segments 1–250, 251–350, and 351–600 matched the 5′ upstream region (10 bp per segment), the TSS to TES region (1% per segment) and the 3′ downstream region (10 bp per segment), respectively. The RM-specific H3K14ac pattern corresponded to incomplete acetylation in segments 251–266 (region immediately downstream of the TSS, termed R1 hereafter) and increased acetylation in segments 284–333 (termed R2 hereafter) (Fig. 2f). To determine the genes contributing to this pattern, we computed, at every segment, the differential profile *d* of H3K14ac between RM and BY. We next computed, for each gene, the difference *δ* between the median of *d* values in R2 and the median of *d* values in R1. As expected, the distribution of *δ* values among genes was skewed toward high values; an upper tail at *δ* > 10 was contributed by 529 genes, where the re-distribution of H3K14ac in RM was the most pronounced. We next extracted, for these genes, the nucleosome-level ChIP-CHIP H3K14ac values of [15] in 60 BY × RM segregants. For each segregant, we constructed a gene-level ‘phenotype’ phen = m2 − m1, where m2 was the mean rank of the segregant over nucleosomes of the second half of the gene body, and m1 was the mean rank of the segregant over nucleosomes of the first half of the gene body. This way, a high ‘phenotypic’ trait corresponded to pronounced imbalance of H3K14ac toward the 3′ end of the gene. A principal component analysis of these 529 phenotypes revealed two significant components, which were then considered as ‘metaphenotypes’ representing the global trend in each segregant. We searched for QTL linked to these metaphenotypes by applying a non-parametric linkage test at every marker position, as previously described [58]. The significance was determined empirically, by running the linkage test on ten permuted datasets.

### Nucleosome-level analysis

Inference of nucleosome positions, their correspondence between strains and the inference of SNEPs were all done using *NucleoMiner2.0*, which are described in detail elsewhere [44]. Briefly, this pipeline called *TemplateFilter* [59] maps nucleosomes in each strain; it applies likelihood ratio test to match nucleosomes and test the reproducibility of their positions, both across biological replicates and across strains; and it uses *DESeq* [57] to detect SNEPs by testing the statistical significance of an interaction term in a generalized linear model fitted to the data.

### Data availability

The raw data is available at Array Express (http://www.ebi.ac.uk/arrayexpress/) under accession numbers E-MTAB-3390 and E-MTAB-2671.

## Additional files

Additional file 1:
**Western blot control experiments. A)** Antibody specificity. Whole cell extracts of wild-type BY strain (black symbol) as well as indicated histone point mutants were probed with antibodies indicated above the gels. **B**) Whole cell extracts from BY, RM and YJM strains (symbols in black, red and green respectively). Additional file 2: List of samples generated and their sequencing depth. Additional file 3:
**Proportion of variance explained by each principal component.** The red line indicates the highest proportion of variance that is expected to be explained by chance only. It was calculated as the proportion of variance explained by the first component obtained on a permuted dataset, and averaged over three permutations. The first four components obtained on the actual data are highly significant. Components 5 and 6 are marginally significant, and all successive ones are non-significant. Additional file 4: Principal Component Analysis performed on ChIP/MNase profiles. Additional file 5: Table of sequence polymorphisms and expression differences of 23 chromatin modifiers among the BY, RM and YJM strains. Additional file 6:
**Normalized coverage profiles along an average gene.** The ChIP coverage values of each histone mark shown in Fig. 2A were divided by the MNase coverage of each strain (in per-million reads, normalized by the size factor of the MNase sample, and averaged across replicates). Colors correspond to strains. Additional file 7: Distances used for gene clustering are not correlated with differences in gene size. For each pair of genes (dots), the gene–gene epigenomic distance that was used for gene clustering (Fig. 3A-B) is indicated on the y-axis. The difference in size between the two genes is indicated on the x-axis. Pairs of genes with identical size were set to x = 1 to allow logarithmic scaling of the axis. Only 10,000 random gene pairs are represented to allow visualization. The Spearman correlation coefficient using these pairs was -0.03. Additional file 8: Inter-strain differential profiles of acetylation along an averaged gene (same binning as for Fig. 3). Additional file 9: Transcription rates (y-axis) reported in [43] for all genes (grey) and for genes belonging to clusters 17, 18 and 19 of Fig. 3. Additional file 10:
**Epigenomic BY-YJM divergence of three sets of genes.** The gene clusters of Fig. 3B, which were defined by similarity in BY–RM divergence, are represented here to display their pattern of BY-YJM divergence. They are displayed in the exact same order as on Fig. 3B, with colors showing the difference between the log_2_(ChIP/MNase) profiles of the BY and YJM strains. Differential gene expression is from [23] in standard rich conditions (YPD medium). Cyan: missing mRNA data. Additional file 11: Table of clusters of genes sharing similar chromatin variation profiles in the RM/YJM comparison (A) or in the BY/YJM comparison (B). Additional file 12: Table of *epidiv* values of all genes. Additional file 13:
**A)** Relative profiles (region from TSS to TES was divided in 1% bins) of inter-strain chromatin divergence of the *LCL2, ATG17, SAG1,* and *NIT1* genes. **B**) Absolute profiles (region from TSS to TES was divided in 10 bp bins) of genes *ISY1* (medium epidiv, no TATA) and *QDR2* (extreme epidiv, TATA). Additional file 14: Epidiv values of all genes as a function of the transcription rate in standard growth conditions, as determined by Miller et al. [43]. Additional file 15:
*Epidiv* dependence on gene size. Additional file 16: Epidiv association with TATA within classes of genes of similar size. Same representation as Fig. 4D, but on subcategories of genes of indicated size range (from TSS to TES). *P* values correspond to Wilcoxon test against the null hypothesis of no epidiv difference between TATA and TATA-less genes. Bars: median values. Additional file 17: Epidiv recalculated using fixed-size physical regions. For each gene, the region spanning 500 bp upstream the TSS to 1,500 bp downstream the TSS was segmented in 10 bp bins and *epidiv w*as calculated using the same anova model and procedure as described in the main text. **A)** Correlation between these recalculated *epidiv* values and transcriptional responsiveness. **B**) Correlation between these recalculated *epidiv* values and presence of a TATA box. Additional file 18: Number of well-positioned and fuzzy nucleosomes mapped in each strain. Additional file 19: Tables of nucleosomal positions in each strain. Additional file 20: Positioning divergence of nucleosomes that are well-positioned in BY (**A**) or in RM (**B**) but did not match a well-positioned nucleosome of YJM. Same representation as Fig. 5B. Numbers in magenta indicate how many of these nucleosomes are considered to be ‘shifted’ between the two strains. Additional file 21: Table of SNEP detection between BY and RM strains. Additional file 22: Table of SNEP detection between BY and YJM strains. Additional file 23: Table of SNEP detection between RM and YJM strains. Additional file 24: Density of SNEPs along an average gene. Curves are colored according to the strain where the mark is more abundant. For example, BY/RM SNEPs for H4K12ac where acetylation is higher in RM correspond to the red curve of the top right panel. Note that, since SNEPs account for MNase-seq inter-strain differences, these profiles do not necessarily correspond to the differences between the ChIP profiles in Fig. 2. Additional file 25:
**Regionality vs. precision of variation relative to the YJM strain.** As in Fig. 7A, regionality of variation of each mark is shown for the BY/YJM (**A**) and for the RM/YJM (**B**) comparisons. **C**) Same as Fig. 7A for H3K4me3, together with a randomization applied only to nucleosomes where the ChIP/MNase ratio is above 1 in at least one strain. Additional file 26: Inter-strain expression changes (y-axis) for all genes (grey dots) and for genes containing a SNEP at nucleosome position +1. Upper panels: BY/RM comparison (using mRNA data from [33]). Lower panels: BY/YJM comparison (using mRNA data from [23]). Dots are colored according to the strain where the epigenetic mark is enriched (black: BY, red: RM, green: YJM). High values on the y-axis correspond to higher mRNA expression in the BY strain. Additional file 27: Similar co-variation of histone marks at isolated vs. regional SNEPs. H3K4me3 SNEPs were termed ‘isolated’ when both flanking nucleosomes did not contain an H3K4me3 SNEP. All others were termed ‘regional’. The same definition was applied to SNEPs of other marks. On each set of nucleosomes (those corresponding to regional and those corresponding to isolated SNEPs for mark (1)), co-variation was quantified as in Fig. 8C, by computing the fraction of BY–RM isolated or regional SNEPs of mark (1) that showed synergistic and significant BY–RM differences in mark (2). Additional file 28: IDs of genes in clusters of shared inter-strain patterns of chromatin variation. Additional file 29: ChIP coverage profiles of the indicated marks along an average gene for each sample (in per-million reads, normalized and averaged across replicates).

## Abbreviations

ANOVA: analysis of variance
ChIP-CHIP: chromatin immunoprecipitation and hybridization on DNA microarrays
ChIP-seq: chromatin immunoprecipitation and sequencing
CUR: common uninterrupted region
FDR: false discovery rate
HDAC: histone de-acetylase complex
PCA: principal component analysis
QTL: quantitative trait locus
aceQTL: quantitative trait locus of chromatin acetylation
eQTL: quantitative trait locus of expression
SAGA: Spt-Ada-Gcn5 acetyltransferase
SNEP: single-nucleosome epi-polymorphism
SNP: single-nucleotide polymorphism
TSA: trichostatin-A
TSS: transcription start site
TES: transcription end site
UNR: unaligned nucleosomal regions

## References

[1] Vaughn MW, Tanurd Ic M, Lippman Z, Jiang H, Carrasquillo R, Rabinowicz PD (2007). Epigenetic natural variation in *Arabidopsis thaliana*. PLoS Biol.

[2] Schilling E, Chartouni CE, Rehli M (2009). Allele-specific DNA methylation in mouse strains is mainly determined by *cis*-acting sequences. Genome Res.

[3] Liu S, Sun K, Jiang T, Ho JP, Liu B, Feng J (2012). Natural epigenetic variation in the female great roundleaf bat (*Hipposideros armiger*) populations. Mol Genet Genomics.

[4] Zhang D, Cheng L, Badner JA, Chen C, Chen Q, Luo W (2010). Genetic control of individual differences in gene-specific methylation in human brain. Am J Hum Genet.

[5] Gibbs JR, van der Brug MP, Hernandez DG, Traynor BJ, Nalls MA, Lai SL (2010). Abundant quantitative trait loci exist for DNA methylation and gene expression in human brain. PLoS Genet.

[6] Nagarajan M, Veyrieras JB, de Dieuleveult M, Bottin H, Fehrmann S, Abraham AL (2010). Natural single-nucleosome epi-polymorphisms in yeast. PLoS Genet.

[7] Kilpinen H, Waszak SM, Gschwind AR, Raghav SK, Witwicki RM, Orioli A (2013). Coordinated effects of sequence variation on DNA binding, chromatin structure, and transcription. Science.

[8] Kasowski M, Kyriazopoulou-Panagiotopoulou S, Grubert F, Zaugg JB, Kundaje A, Liu Y (2013). Extensive variation in chromatin states across humans. Science.

[9] McVicker G, van de Geijn B, Degner JF, Cain CE, Banovich NE, Raj A (2013). Identification of Genetic variants that affect histone modifications in human cells. Science.

[10] McDaniell R, Lee BK, Song L, Liu Z, Boyle AP, Erdos MR (2010). Heritable individual-specific and allele-specific chromatin signatures in humans. Science.

[11] Chai X, Nagarajan S, Kim K, Lee K, Choi JK (2013). Regulation of the boundaries of accessible chromatin. PLoS Genet.

[12] Moghaddam AMB, Roudier F, Seifert M, Bérard C, Magniette M-LM, Ashtiyani RK (2011). Additive inheritance of histone modifications in *Arabidopsis thaliana* intra-specific hybrids. Plant J.

[13] Rintisch C, Heinig M, Bauerfeind A, Schafer S, Mieth C, Patone G (2014). Natural variation of histone modification and its impact on gene expression in the rat genome. Genome Res.

[14] Kadota M, Yang HH, Hu N, Wang C, Hu Y, Taylor PR (2007). Allele-specific chromatin immunoprecipitation studies show genetic influence on chromatin state in human genome. PLoS Genet.

[15] Abraham AL, Nagarajan M, Veyrieras JB, Bottin H, Steinmetz LM, Yvert G (2012). Genetic modifiers of chromatin acetylation antagonize the reprogramming of epi-polymorphisms. PLoS Genet.

[16] Liu CL, Kaplan T, Kim M, Buratowski S, Schreiber SL, Friedman N (2005). Single-nucleosome mapping of histone modifications in *S. cerevisiae*. PLoS Biol.

[17] Pokholok DK, Harbison CT, Levine S, Cole M, Hannett NM, Lee TI (2005). Genome-wide map of nucleosome acetylation and methylation in yeast. Cell.

[18] Chabbert CD, Adjalley SH, Klaus B, Fritsch ES, Gupta I, Pelechano V (2015). A high-throughput ChIP-Seq for large-scale chromatin studies. Mol Syst Biol.

[19] Wei W, McCusker JH, Hyman RW, Jones T, Ning Y, Cao Z (2007). Genome sequencing and comparative analysis of *Saccharomyces cerevisiae* strain YJM789. Proc Natl Acad Sci USA.

[20] Liti G, Carter DM, Moses AM, Warringer J, Parts L, James SA (2009). Population genomics of domestic and wild yeasts. Nature.

[21] Brem RB, Yvert G, Clinton R, Kruglyak L (2002). Genetic dissection of transcriptional regulation in budding yeast. Science.

[22] Ruderfer DM, Pratt SC, Seidel HS, Kruglyak L (2006). Population genomic analysis of outcrossing and recombination in yeast. Nat Genet.

[23] Gagneur J, Sinha H, Perocchi F, Bourgon R, Huber W, Steinmetz LM (2009). Genome-wide allele- and strand-specific expression profiling. Mol Syst Biol.

[24] Dai J, Hyland EM, Yuan DS, Huang H, Bader JS, Boeke JD (2008). Probing nucleosome function: a highly versatile library of synthetic histone H3 and H4 mutants. Cell.

[25] Breunig JS, Hackett SR, Rabinowitz JD, Kruglyak L (2014). Genetic basis of metabolome variation in yeast. PLoS Genet.

[26] Mews P, Zee BM, Liu S, Donahue G, Garcia BA, Berger SL (2014). Histone methylation has dynamics distinct from those of histone acetylation in cell cycle reentry from quiescence. Mol Cell Biol.

[27] Kuo MH, Brownell JE, Sobel RE, Ranalli TA, Cook RG, Edmondson DG (1996). Transcription-linked acetylation by Gcn5p of histones H3 and H4 at specific lysines. Nature.

[28] Rosaleny LE, Ruiz-García AB, García-Martínez J, Pérez-Ortín JE, Tordera V (2007). The Sas3p and Gcn5p histone acetyltransferases are recruited to similar genes. Genome Biol.

[29] Vicente-Muñoz S, Romero P, Magraner-Pardo L, Martinez-Jimenez CP, Tordera V, Pamblanco M (2014). Comprehensive analysis of interacting proteins and genome-wide location studies of the Sas3-dependent NuA3 histone acetyltransferase complex. FEBS Open Bio.

[30] Robyr D, Suka Y, Xenarios I, Kurdistani SK, Wang A, Suka N (2002). Microarray deacetylation maps determine genome-wide functions for yeast histone deacetylases. Cell.

[31] Li S, Shogren-Knaak MA (2009). The Gcn5 bromodomain of the SAGA complex facilitates cooperative and cross-tail acetylation of nucleosomes. J Biol Chem.

[32] Cieniewicz AM, Moreland L, Ringel AE, Mackintosh SG, Raman A, Gilbert TM, et al. The bromodomain of Gcn5 regulates site-specificity of lysine acetylation on histone H3. Mol Cell Proteomics. 2014:mcp.M114.038174. 10.1074/mcp.M114.038174 PMC4223480 25106422

[33] Smith EN, Kruglyak L (2008). Gene–environment interaction in yeast gene expression. PLoS Biol.

[34] Mosesson Y, Voichek Y, Barkai N (2014). Divergence and selectivity of expression-coupled histone modifications in budding yeasts. PLoS One.

[35] Churchman LS, Weissman JS (2011). Nascent transcript sequencing visualizes transcription at nucleotide resolution. Nature.

[36] Schwalb B, Schulz D, Sun M, Zacher B, Dümcke S, Martin DE (2012). Measurement of genome-wide RNA synthesis and decay rates with dynamic transcriptome analysis (DTA). Bioinformatics.

[37] Jónsson ZO, Jha S, Wohlschlegel JA, Dutta A (2004). Rvb1p/Rvb2p recruit Arp5p and assemble a functional Ino80 chromatin remodeling complex. Mol Cell.

[38] Vargas RC, Tenreiro S, Teixeira MC, Fernandes AR, Sa-Correia I (2004). *Saccharomyces cerevisiae* multidrug transporter Qdr2p (Yil121wp): localization and function as a quinidine resistance determinant. Antimicrob Agents Chemother.

[39] Pace HC, Hodawadekar SC, Draganescu A, Huang J, Bieganowski P, Pekarsky Y (2000). Crystal structure of the worm NitFhit Rosetta stone protein reveals a Nit tetramer binding two Fhit dimers. Curr Biol.

[40] Wall DP, Hirsh AE, Fraser HB, Kumm J, Giaever G, Eisen MB (2005). Functional genomic analysis of the rates of protein evolution. Proc Natl Acad Sci USA.

[41] Landry CR, Lemos B, Rifkin SA, Dickinson WJ, Hartl DL (2007). Genetic properties influencing the evolvability of gene expression. Science.

[42] Tirosh I, Weinberger A, Carmi M, Barkai N (2006). A genetic signature of interspecies variations in gene expression. Nat Genet.

[43] Miller C, Schwalb B, Maier K, Schulz D, Dumcke S, Zacher B (2011). Dynamic transcriptome analysis measures rates of mRNA synthesis and decay in yeast. Mol Syst Biol.

[44] Chuffart F, Filleton F, Yvert G. NucleoMiner 2.0: detecting intra-species quantitative epigenomic variation at single-nucleosome resolution. Submitted.

[45] Jansen A, Verstrepen KJ (2011). Nucleosome positioning in *Saccharomyces cerevisiae*. Microbiol Mol Biol Rev.

[46] MacIsaac KD, Wang T, Gordon DB, Gifford DK, Stormo GD, Fraenkel E (2006). An improved map of conserved regulatory sites for *Saccharomyces cerevisiae*. BMC Bioinform.

[47] Hughes AL, Jin Y, Rando OJ, Struhl K (2012). A functional evolutionary approach to identify determinants of nucleosome positioning: a unifying model for establishing the genome-wide pattern. Mol Cell.

[48] Yen K, Vinayachandran V, Batta K, Koerber RT, Pugh BF (2012). Genome-wide nucleosome specificity and directionality of chromatin remodelers. Cell.

[49] Ronald J, Brem RB, Whittle J, Kruglyak L (2005). Local regulatory variation in *Saccharomyces cerevisiae*. PLoS Genet.

[50] Ginsburg DS, Anlembom TE, Wang J, Patel SR, Li B, Hinnebusch AG (2014). NuA4 links methylation of histone H3 lysines 4 and 36 to acetylation of histones H4 and H3. J Biol Chem.

[51] Maltby VE, Martin BJE, Brind’Amour J, Chruscicki AT, McBurney KL, Schulze JM (2012). Histone H3K4 demethylation is negatively regulated by histone H3 acetylation in *Saccharomyces cerevisiae*. Proc Natl Acad Sci.

[52] Brachmann CB, Davies A, Cost GJ, Caputo E, Li J, Hieter P (1998). Designer deletion strains derived from *Saccharomyces cerevisiae* S288C: a useful set of strains and plasmids for PCR-mediated gene disruption and other applications. Yeast.

[53] Langmead B, Salzberg SL (2012). Fast gapped-read alignment with Bowtie 2. Nat Methods.

[54] Li H, Handsaker B, Wysoker A, Fennell T, Ruan J, Homer N (2009). The sequence alignment/map format and SAMtools. Bioinformatics.

[55] Quinlan AR, Hall IM (2010). BEDTools: a flexible suite of utilities for comparing genomic features. Bioinformatics.

[56] Kurtz S, Phillippy A, Delcher AL, Smoot M, Shumway M, Antonescu C (2004). Versatile and open software for comparing large genomes. Genome Biol.

[57] Anders S, Huber W (2010). Differential expression analysis for sequence count data. Genome Biol.

[58] Nogami S, Ohya Y, Yvert G (2007). Genetic complexity and quantitative trait loci mapping of yeast morphological traits. PLoS Genet.

[59] Weiner A, Hughes A, Yassour M, Rando OJ, Friedman N (2010). High-resolution nucleosome mapping reveals transcription-dependent promoter packaging. Genome Res.

[60] Tirosh I, Barkai N (2008). Two strategies for gene regulation by promoter nucleosomes. Genome Res.

