# Supplementary figures and images for: The complex pattern of epigenomic variation between natural yeast strains at single-nucleosome resolution
Source: Epigenetics Chromatin. 2015 Jul 31;8:26. doi: 10.1186/s13072-015-0019-3 (PMC4520285; doi:10.1186/s13072-015-0019-3)

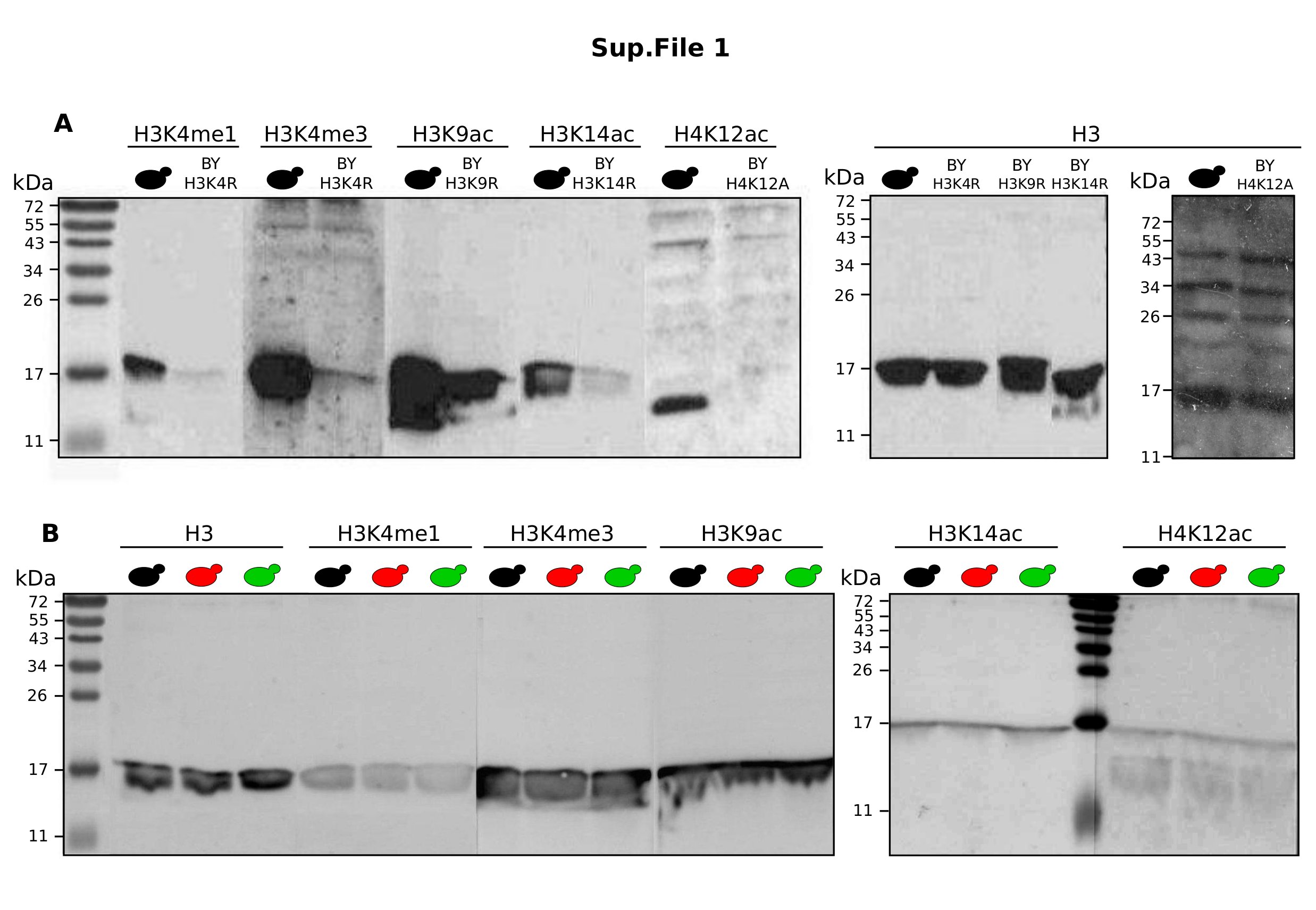

Supplement: Additional file 1: — Western blot control experiments. A) Antibody specificity. Whole cell extracts of wild-type BY strain (black symbol) as well as indicated histone point mutants were probed with antibodies indicated above the gels. B) Whole cell extracts from BY, RM and YJM strains (symbols in black, red and green respectively). [file 13072_2015_19_MOESM1_ESM.jpeg]

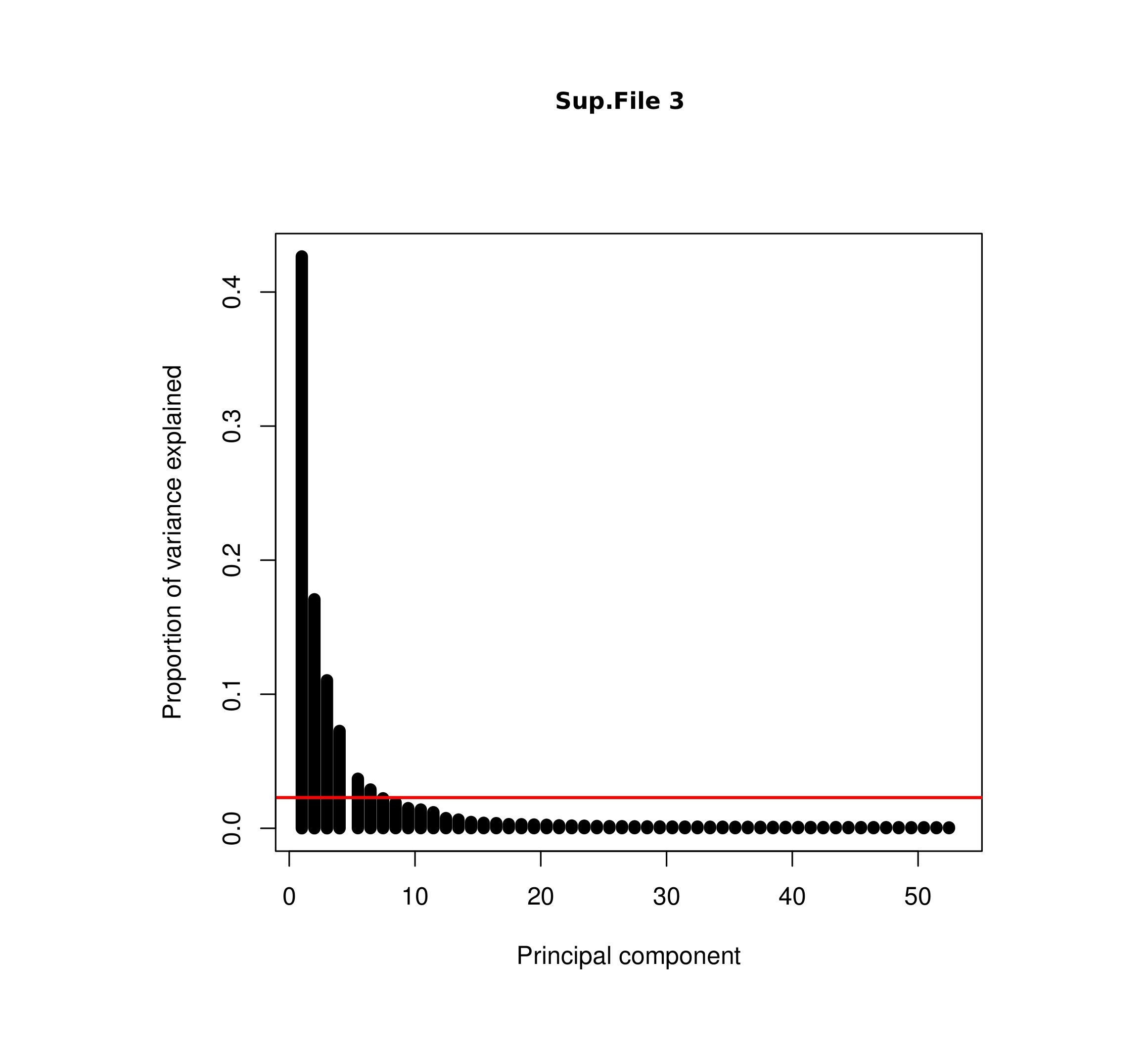

Supplement: Additional file 3: — Proportion of variance explained by each principal component. The red line indicates the highest proportion of variance that is expected to be explained by chance only. It was calculated as the proportion of variance explained by the first component obtained on a permuted dataset, and averaged over three permutations. The first four components obtained on the actual data are highly significant. Components 5 and 6 are marginally significant, and all successive ones are non-significant. [file 13072_2015_19_MOESM3_ESM.jpeg]

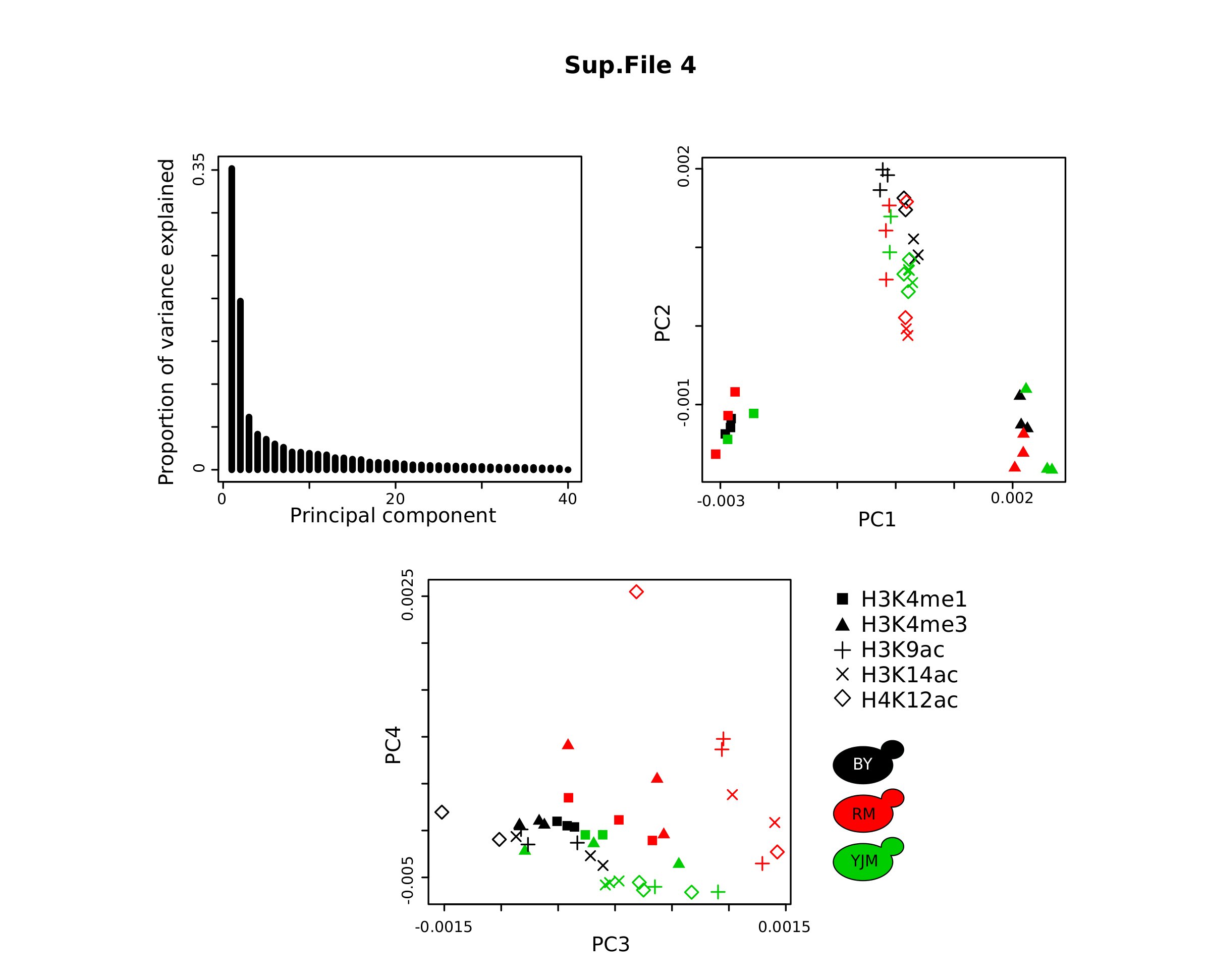

Supplement: Additional file 4: — Principal Component Analysis performed on ChIP/MNase profiles. [file 13072_2015_19_MOESM4_ESM.jpeg]

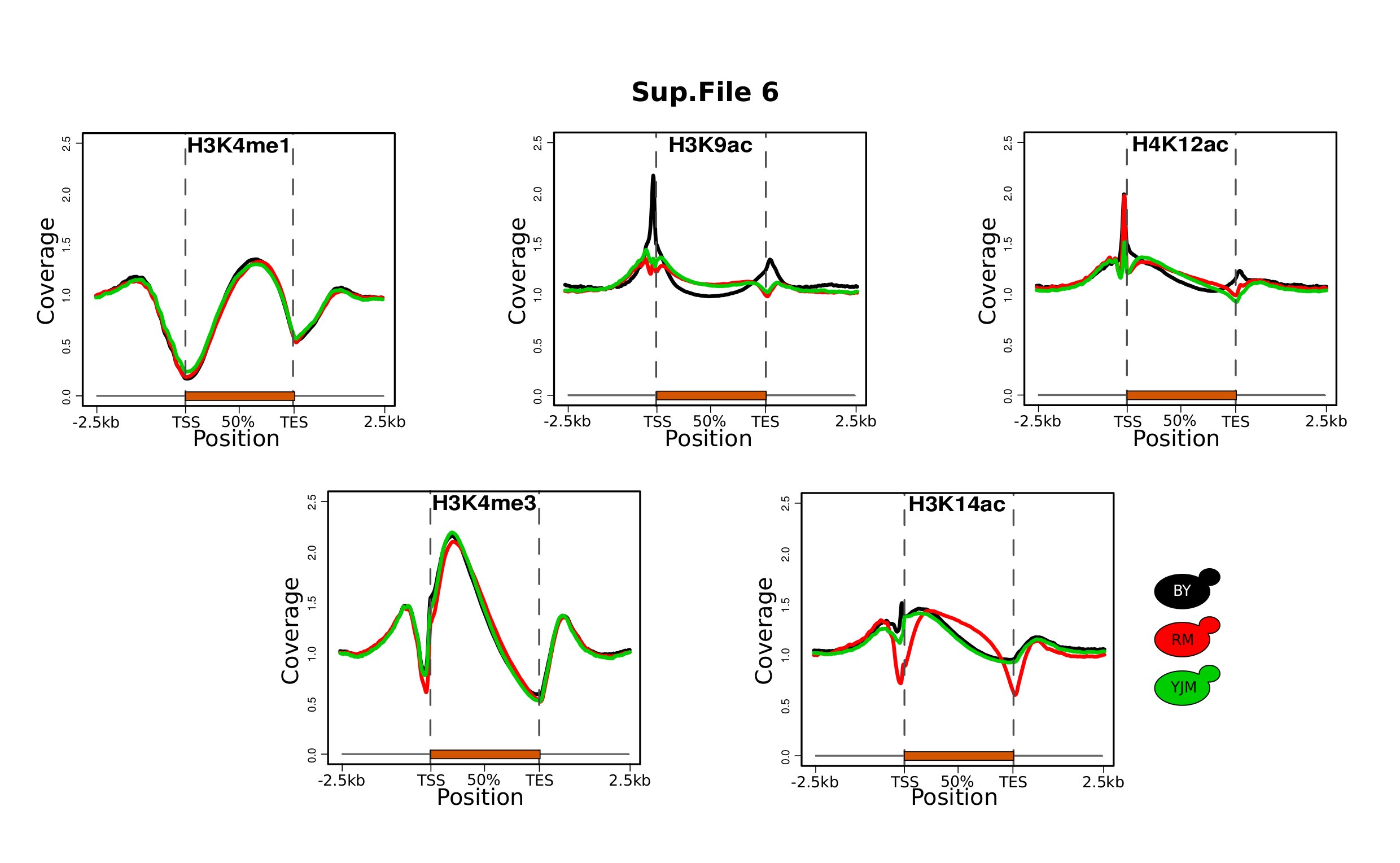

Supplement: Additional file 6: — Normalized coverage profiles along an average gene. The ChIP coverage values of each histone mark shown in Fig. 2A were divided by the MNase coverage of each strain (in per-million reads, normalized by the size factor of the MNase sample, and averaged across replicates). Colors correspond to strains. [file 13072_2015_19_MOESM6_ESM.jpeg]

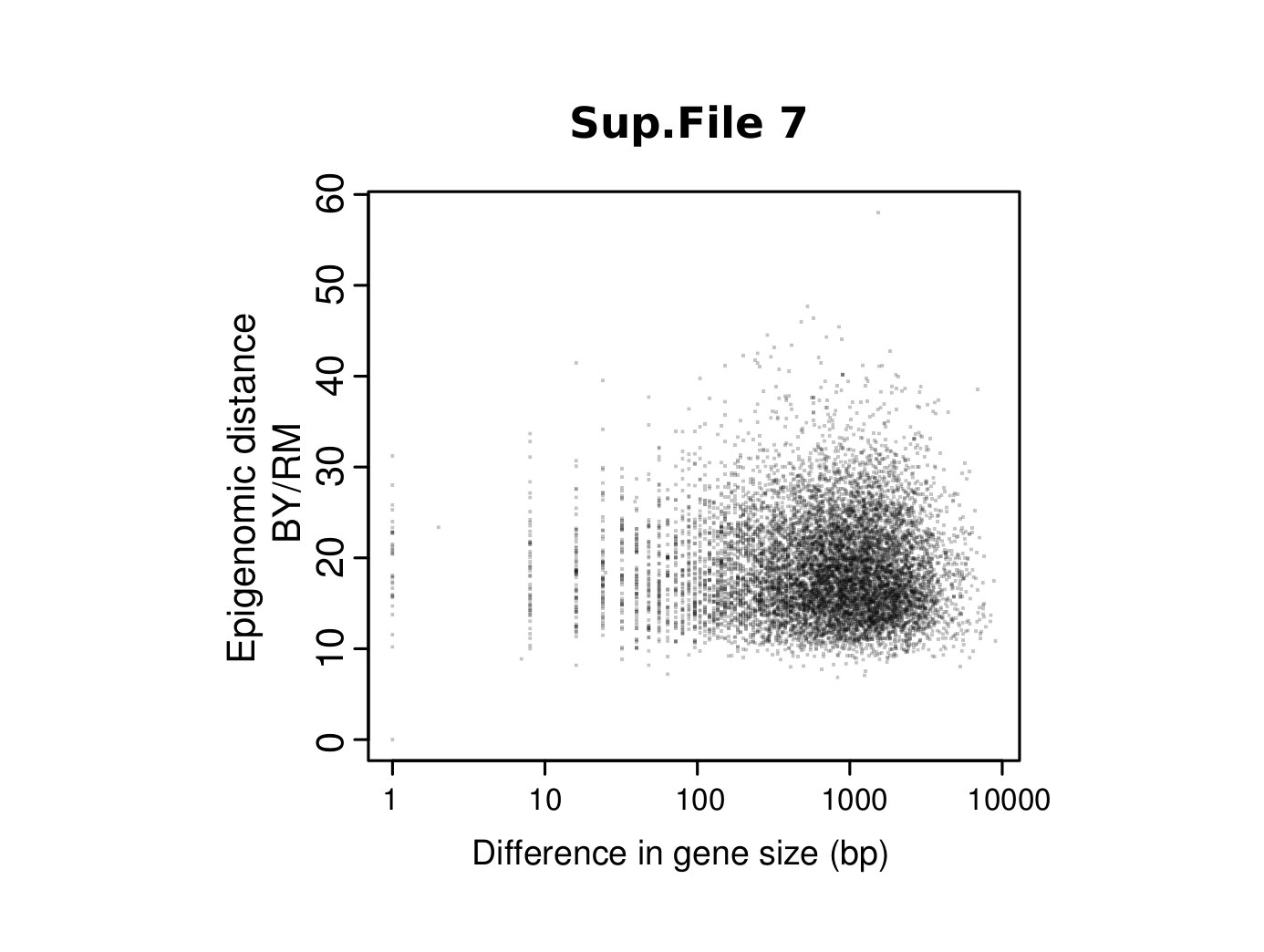

Supplement: Additional file 7: — Distances used for gene clustering are not correlated with differences in gene size. For each pair of genes (dots), the gene–gene epigenomic distance that was used for gene clustering (Fig. 3A-B) is indicated on the y-axis. The difference in size between the two genes is indicated on the x-axis. Pairs of genes with identical size were set to x = 1 to allow logarithmic scaling of the axis. Only 10,000 random gene pairs are represented to allow visualization. The Spearman correlation coefficient using these pairs was -0.03. [file 13072_2015_19_MOESM7_ESM.jpeg]

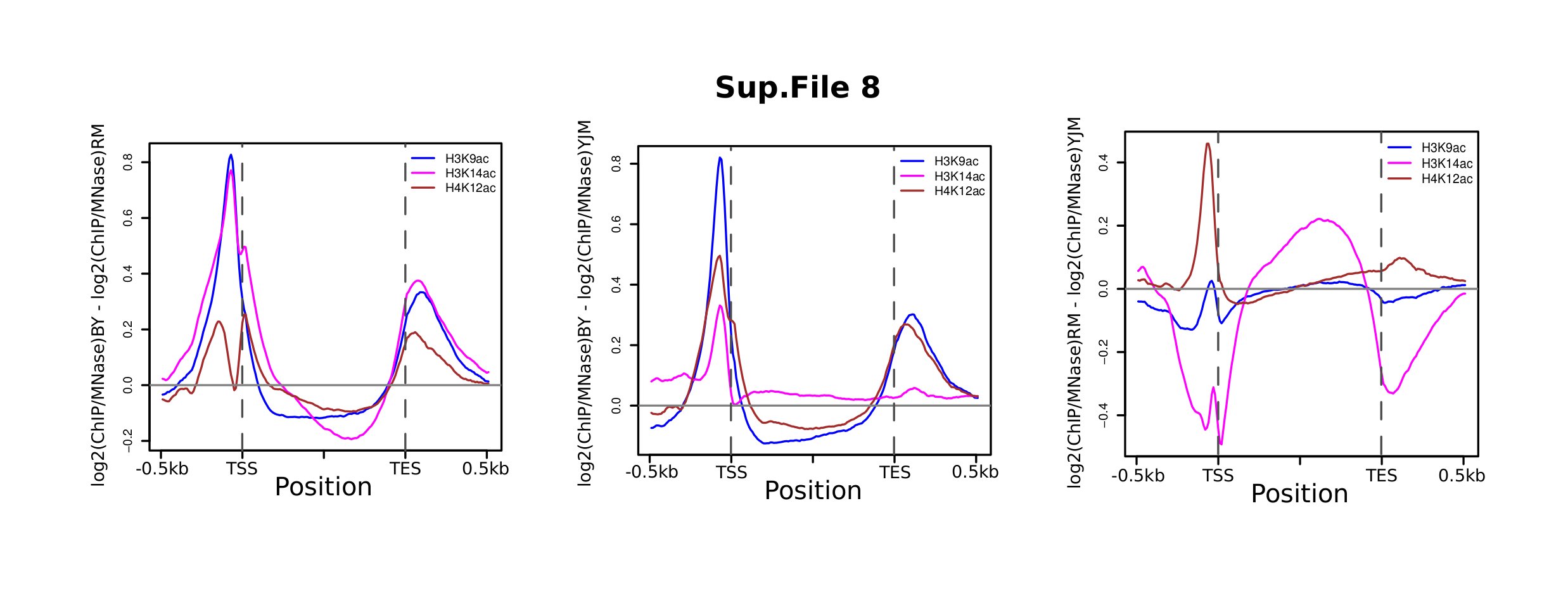

Supplement: Additional file 8: — Inter-strain differential profiles of acetylation along an averaged gene (same binning as for Fig. 3). [file 13072_2015_19_MOESM8_ESM.jpeg]

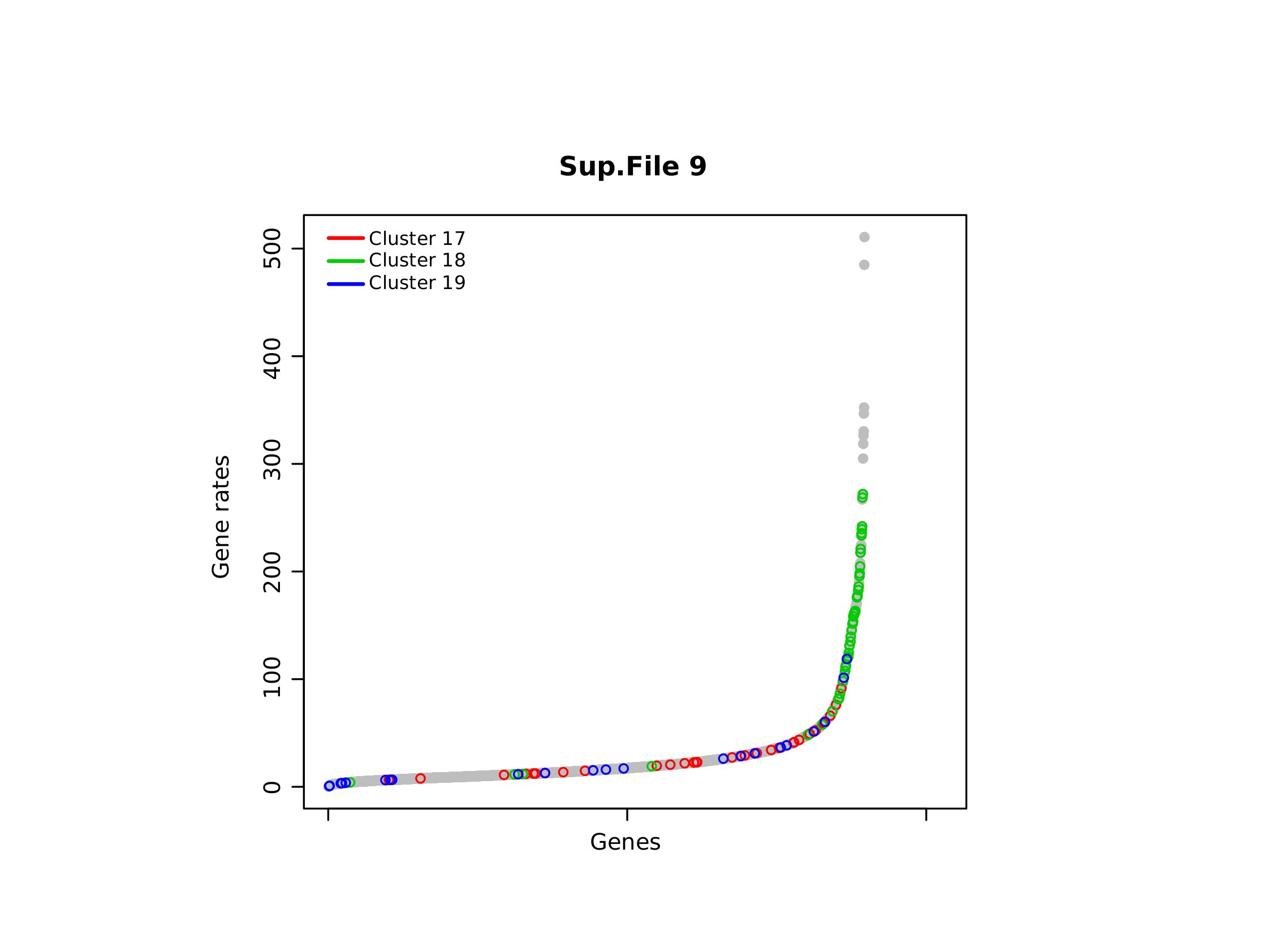

Supplement: Additional file 9: — Transcription rates (y-axis) reported in [43] for all genes (grey) and for genes belonging to clusters 17, 18 and 19 of Fig. 3. [file 13072_2015_19_MOESM9_ESM.jpeg]

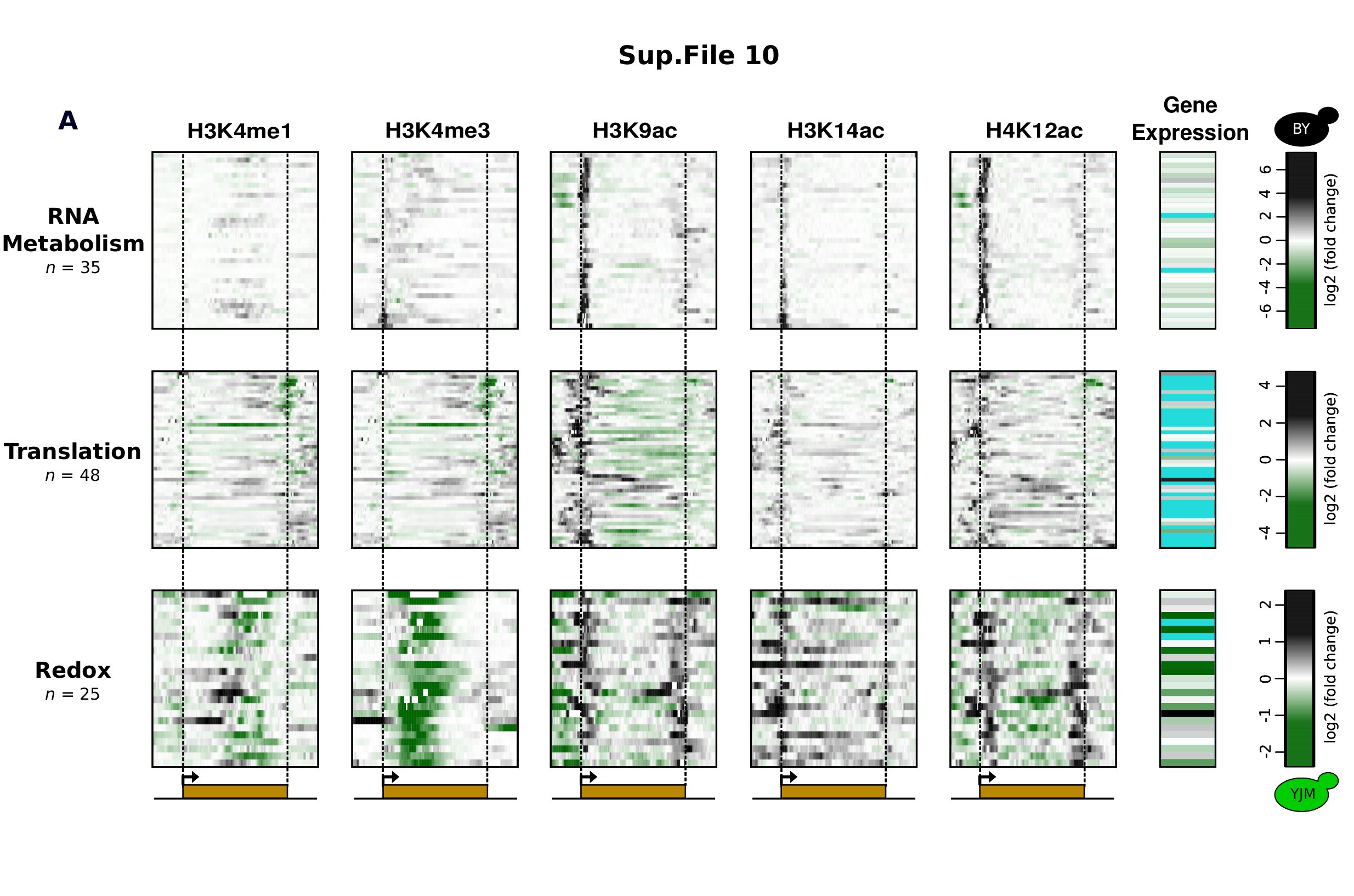

Supplement: Additional file 10: — Epigenomic BY-YJM divergence of three sets of genes. The gene clusters of Fig. 3B, which were defined by similarity in BY–RM divergence, are represented here to display their pattern of BY-YJM divergence. They are displayed in the exact same order as on Fig. 3B, with colors showing the difference between the log2(ChIP/MNase) profiles of the BY and YJM strains. Differential gene expression is from [23] in standard rich conditions (YPD medium). Cyan: missing mRNA data. [file 13072_2015_19_MOESM10_ESM.jpeg]

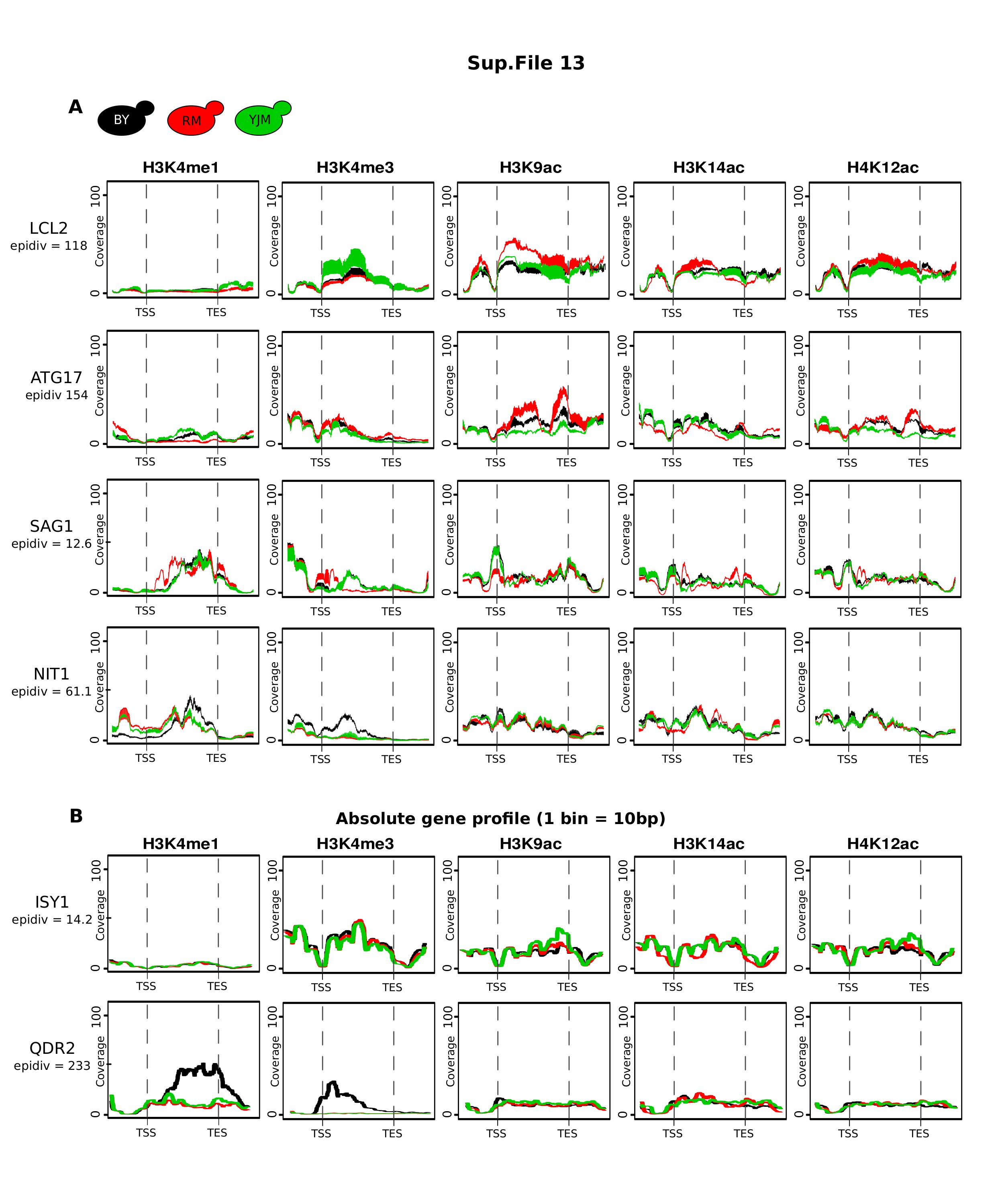

Supplement: Additional file 13: — A) Relative profiles (region from TSS to TES was divided in 1% bins) of inter-strain chromatin divergence of the LCL2, ATG17, SAG1, and NIT1 genes. B) Absolute profiles (region from TSS to TES was divided in 10 bp bins) of genes ISY1 (medium epidiv, no TATA) and QDR2 (extreme epidiv, TATA). [file 13072_2015_19_MOESM13_ESM.jpeg]

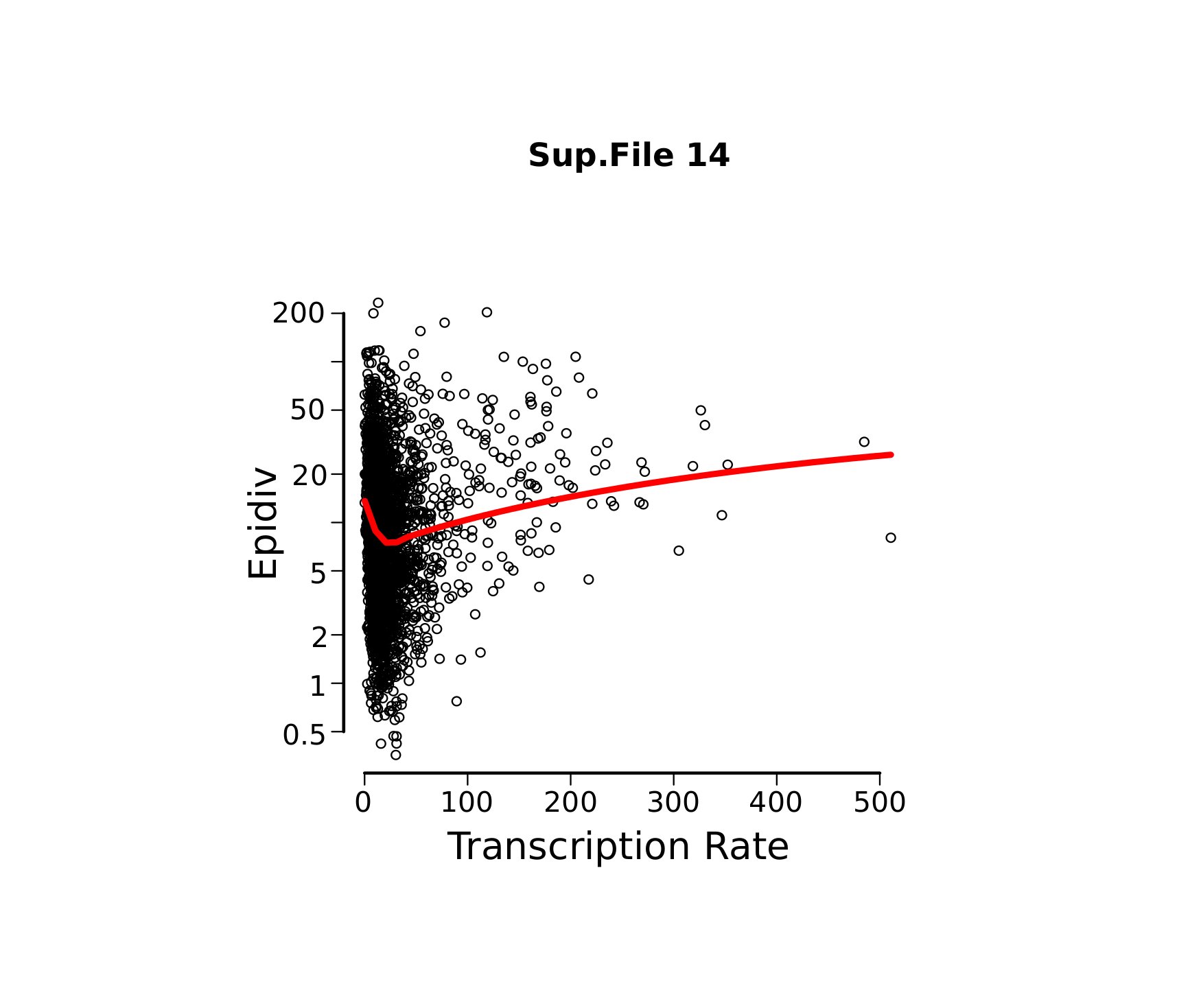

Supplement: Additional file 14: — Epidiv values of all genes as a function of the transcription rate in standard growth conditions, as determined by Miller et al. [43]. [file 13072_2015_19_MOESM14_ESM.jpeg]

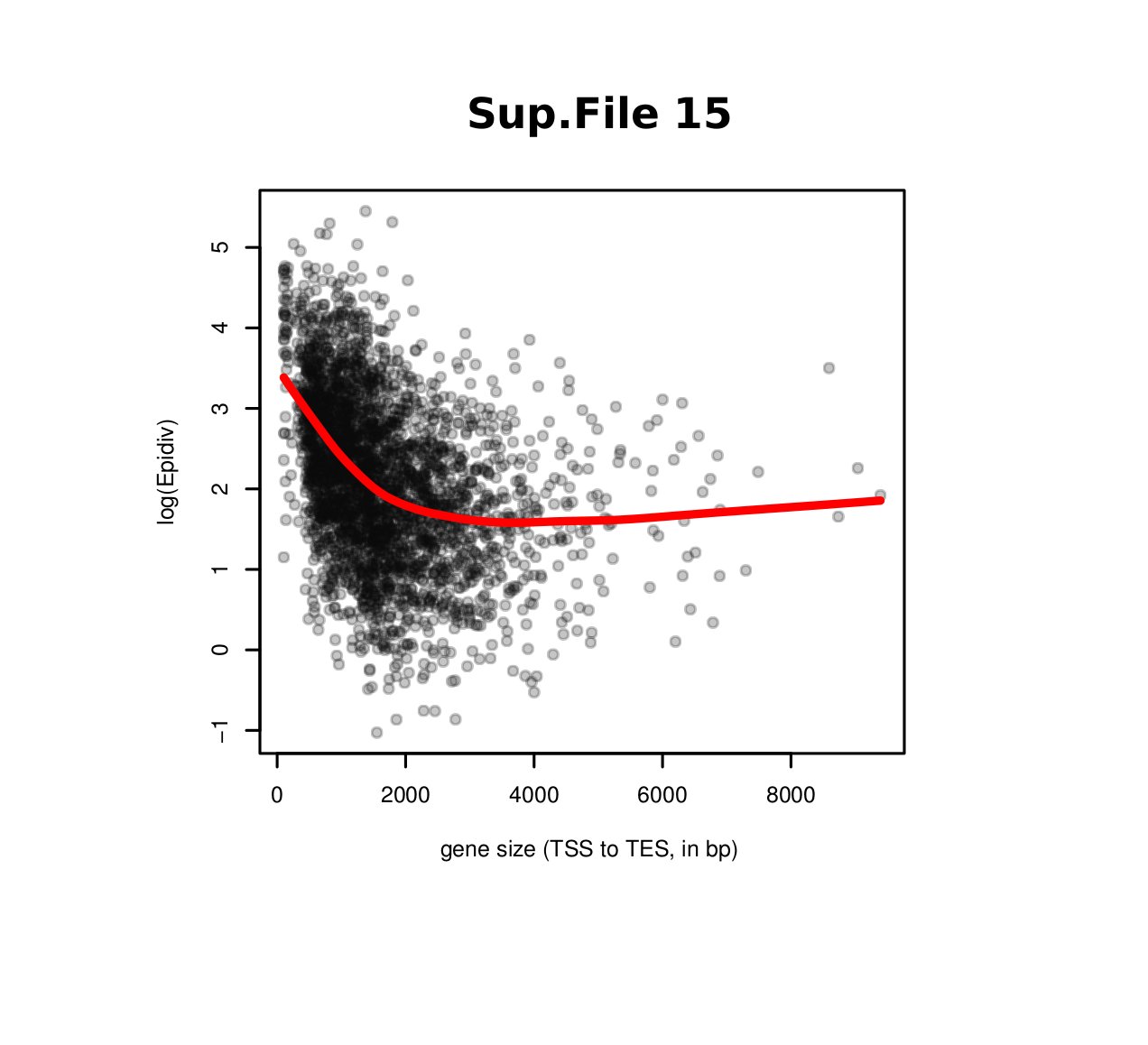

Supplement: Additional file 15: — Epidiv dependence on gene size. [file 13072_2015_19_MOESM15_ESM.jpeg]

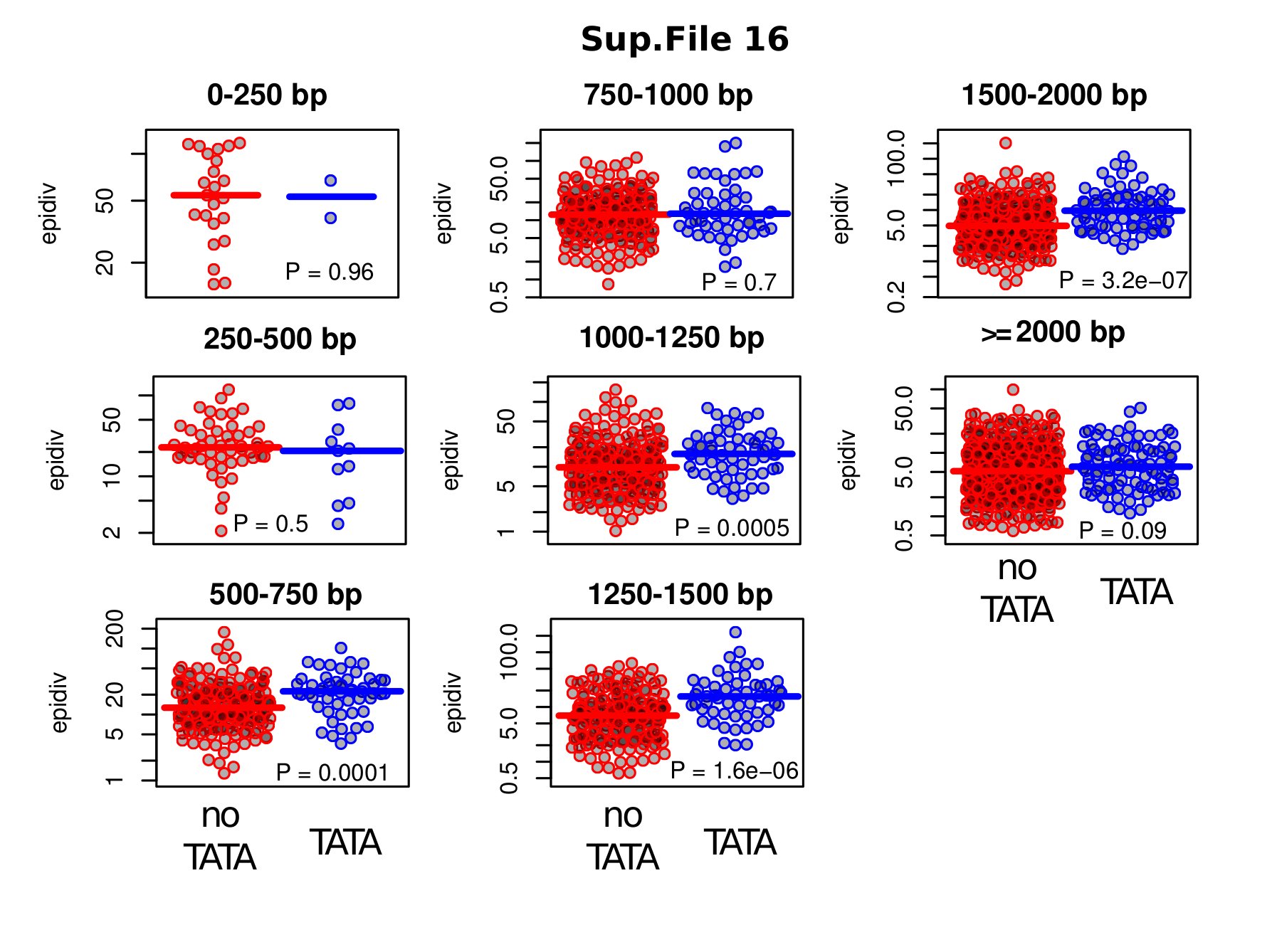

Supplement: Additional file 16: — Epidiv association with TATA within classes of genes of similar size. Same representation as Fig. 4D, but on subcategories of genes of indicated size range (from TSS to TES). P values correspond to Wilcoxon test against the null hypothesis of no epidiv difference between TATA and TATA-less genes. Bars: median values. [file 13072_2015_19_MOESM16_ESM.jpeg]

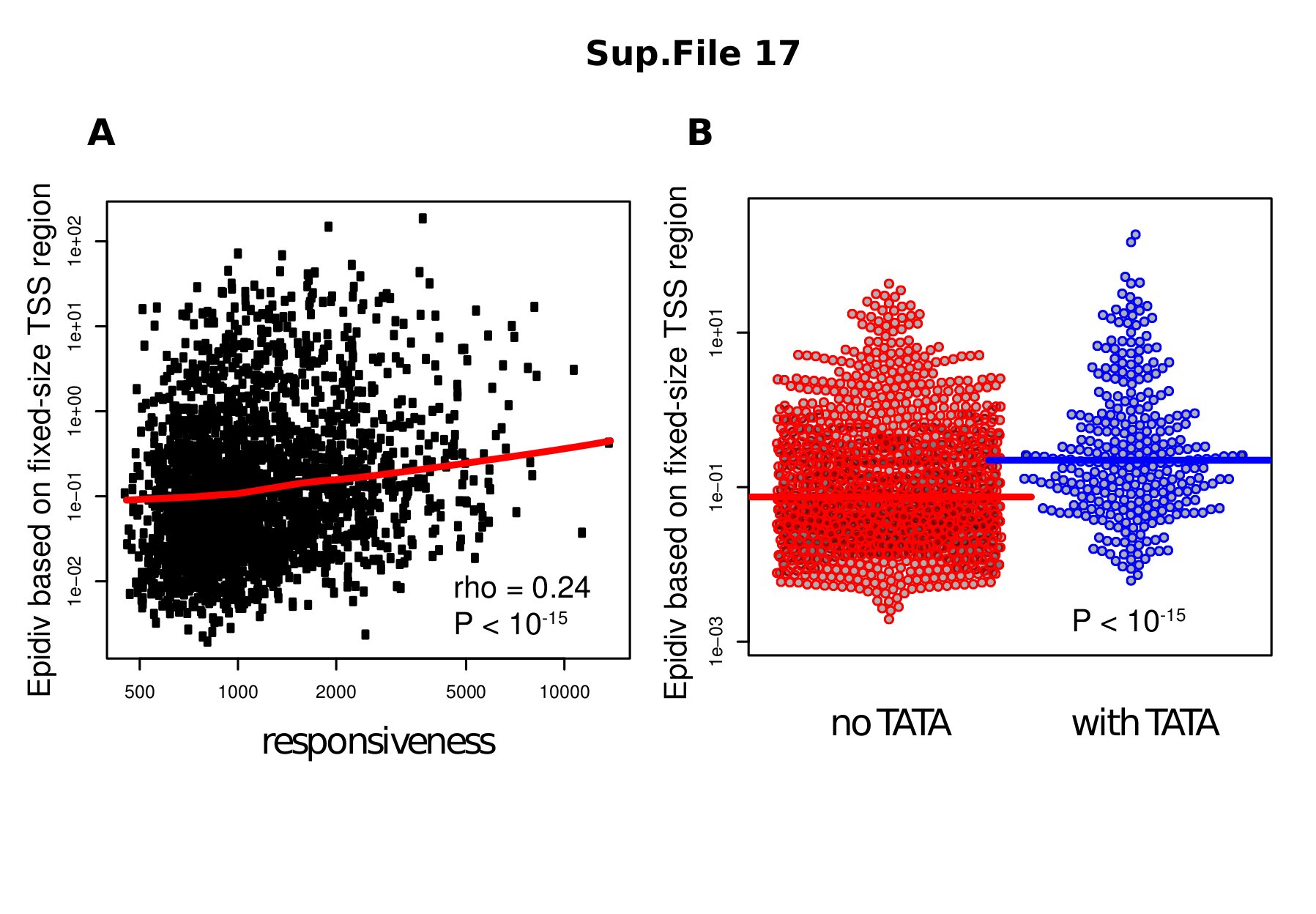

Supplement: Additional file 17: — Epidiv recalculated using fixed-size physical regions. For each gene, the region spanning 500 bp upstream the TSS to 1,500 bp downstream the TSS was segmented in 10 bp bins and epidiv was calculated using the same anova model and procedure as described in the main text. A) Correlation between these recalculated epidiv values and transcriptional responsiveness. B) Correlation between these recalculated epidiv values and presence of a TATA box. [file 13072_2015_19_MOESM17_ESM.jpeg]

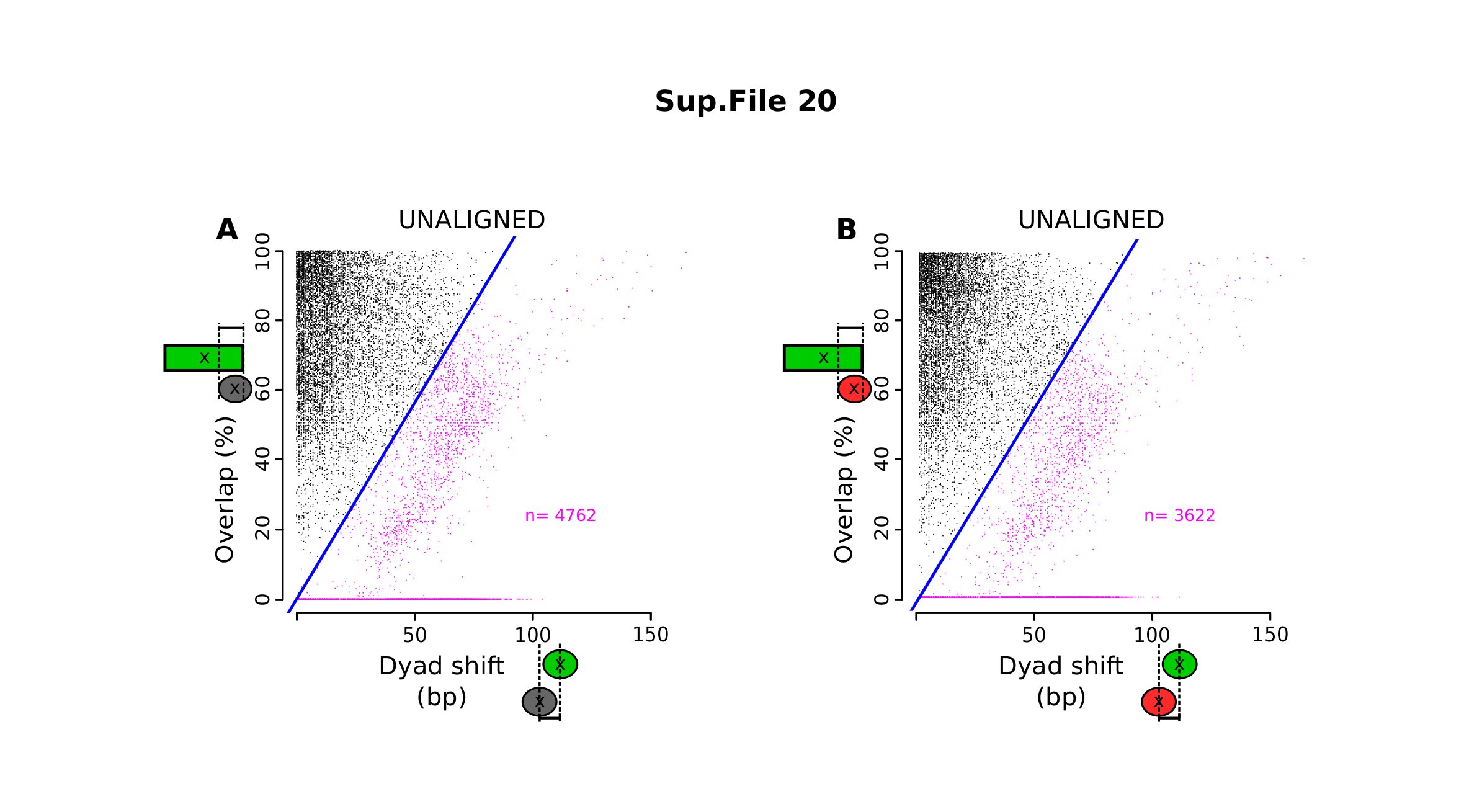

Supplement: Additional file 20: — Positioning divergence of nucleosomes that are well-positioned in BY (A) or in RM (B) but did not match a well-positioned nucleosome of YJM. Same representation as Fig. 5B. Numbers in magenta indicate how many of these nucleosomes are considered to be ‘shifted’ between the two strains. [file 13072_2015_19_MOESM20_ESM.jpeg]

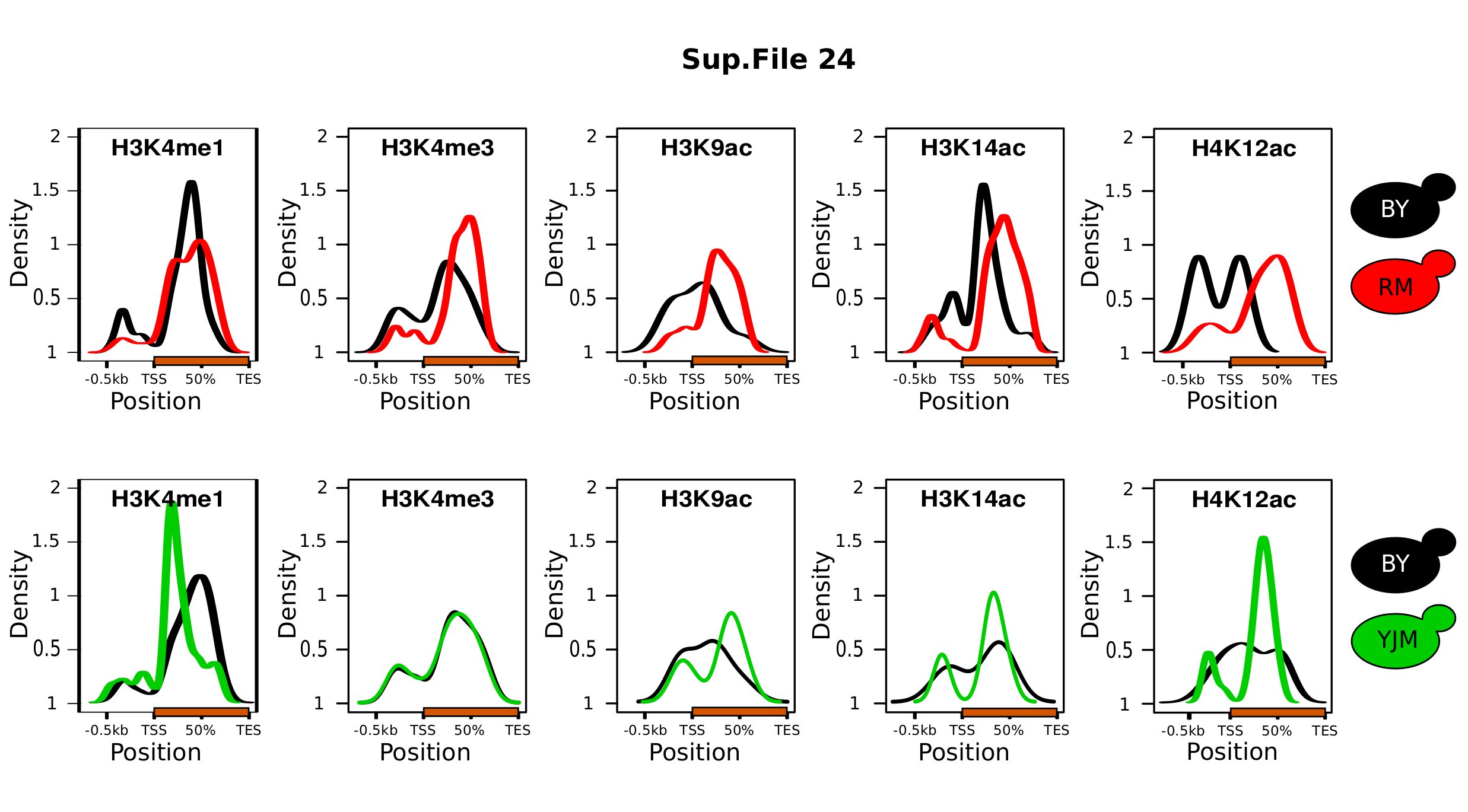

Supplement: Additional file 24: — Density of SNEPs along an average gene. Curves are colored according to the strain where the mark is more abundant. For example, BY/RM SNEPs for H4K12ac where acetylation is higher in RM correspond to the red curve of the top right panel. Note that, since SNEPs account for MNase-seq inter-strain differences, these profiles do not necessarily correspond to the differences between the ChIP profiles in Fig. 2. [file 13072_2015_19_MOESM24_ESM.jpeg]

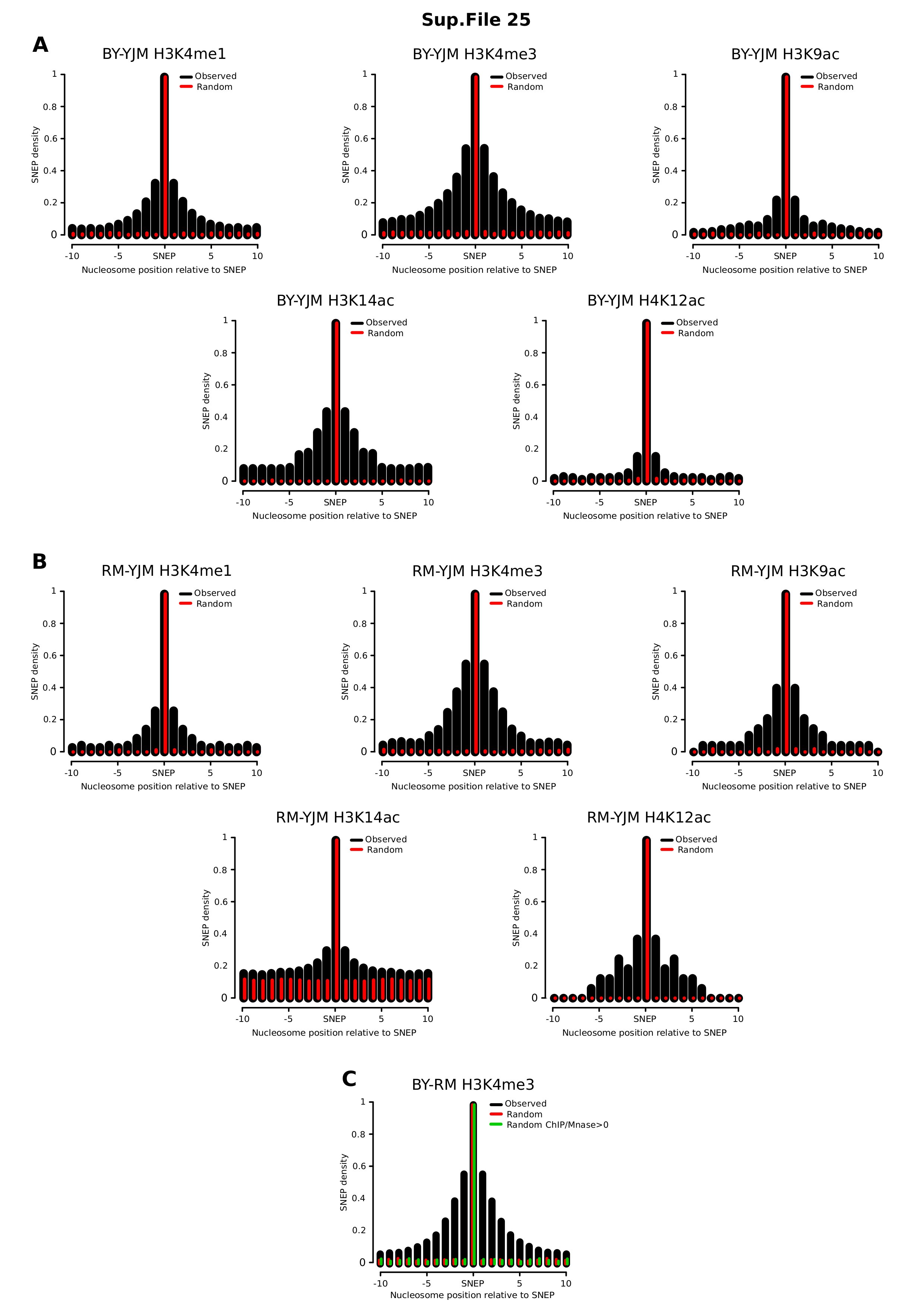

Supplement: Additional file 25: — Regionality vs. precision of variation relative to the YJM strain. As in Fig. 7A, regionality of variation of each mark is shown for the BY/YJM (A) and for the RM/YJM (B) comparisons. C) Same as Fig. 7A for H3K4me3, together with a randomization applied only to nucleosomes where the ChIP/MNase ratio is above 1 in at least one strain. [file 13072_2015_19_MOESM25_ESM.jpeg]

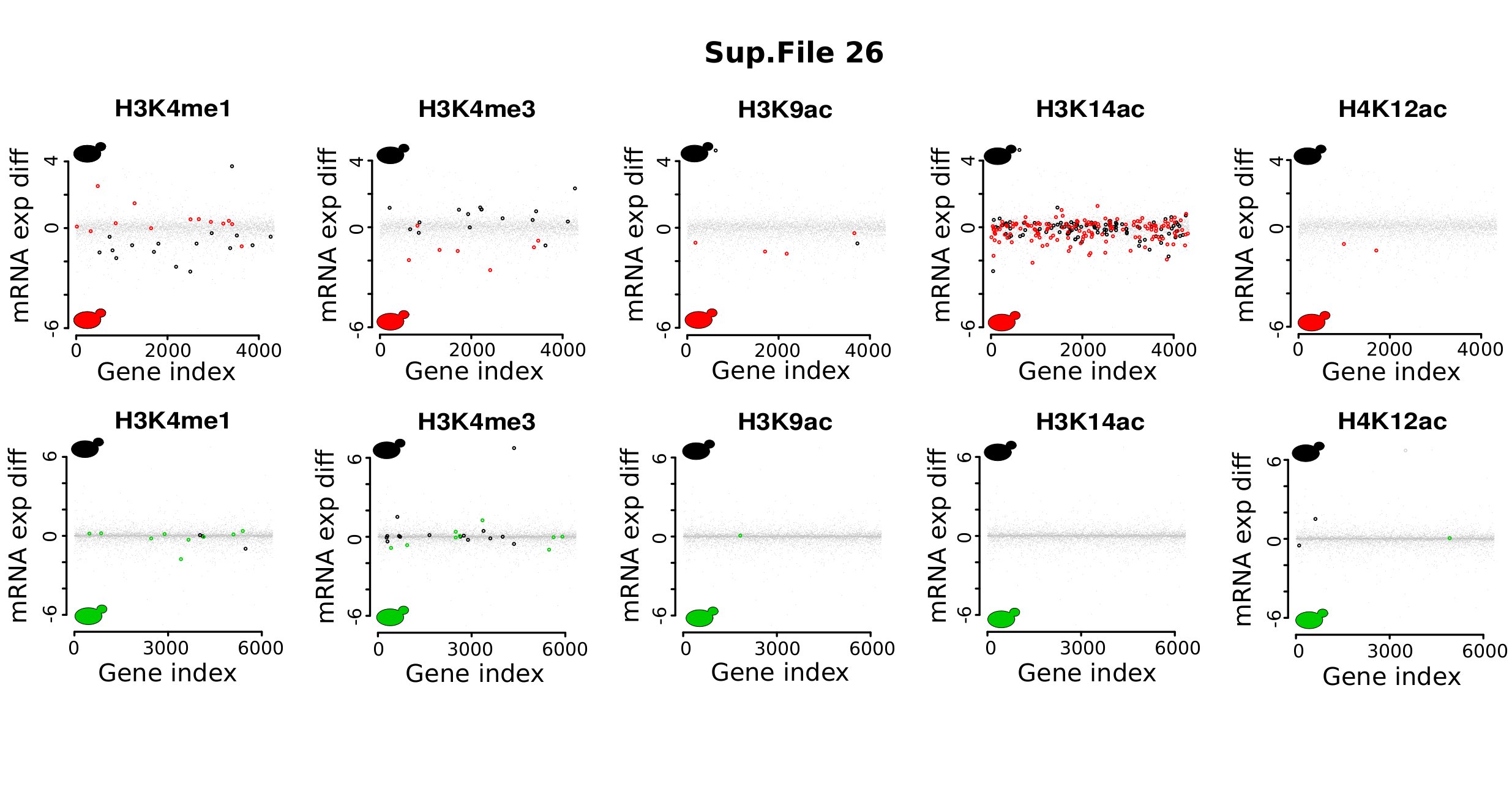

Supplement: Additional file 26: — Inter-strain expression changes (y-axis) for all genes (grey dots) and for genes containing a SNEP at nucleosome position +1. Upper panels: BY/RM comparison (using mRNA data from [33]). Lower panels: BY/YJM comparison (using mRNA data from [23]). Dots are colored according to the strain where the epigenetic mark is enriched (black: BY, red: RM, green: YJM). High values on the y-axis correspond to higher mRNA expression in the BY strain. [file 13072_2015_19_MOESM26_ESM.jpeg]

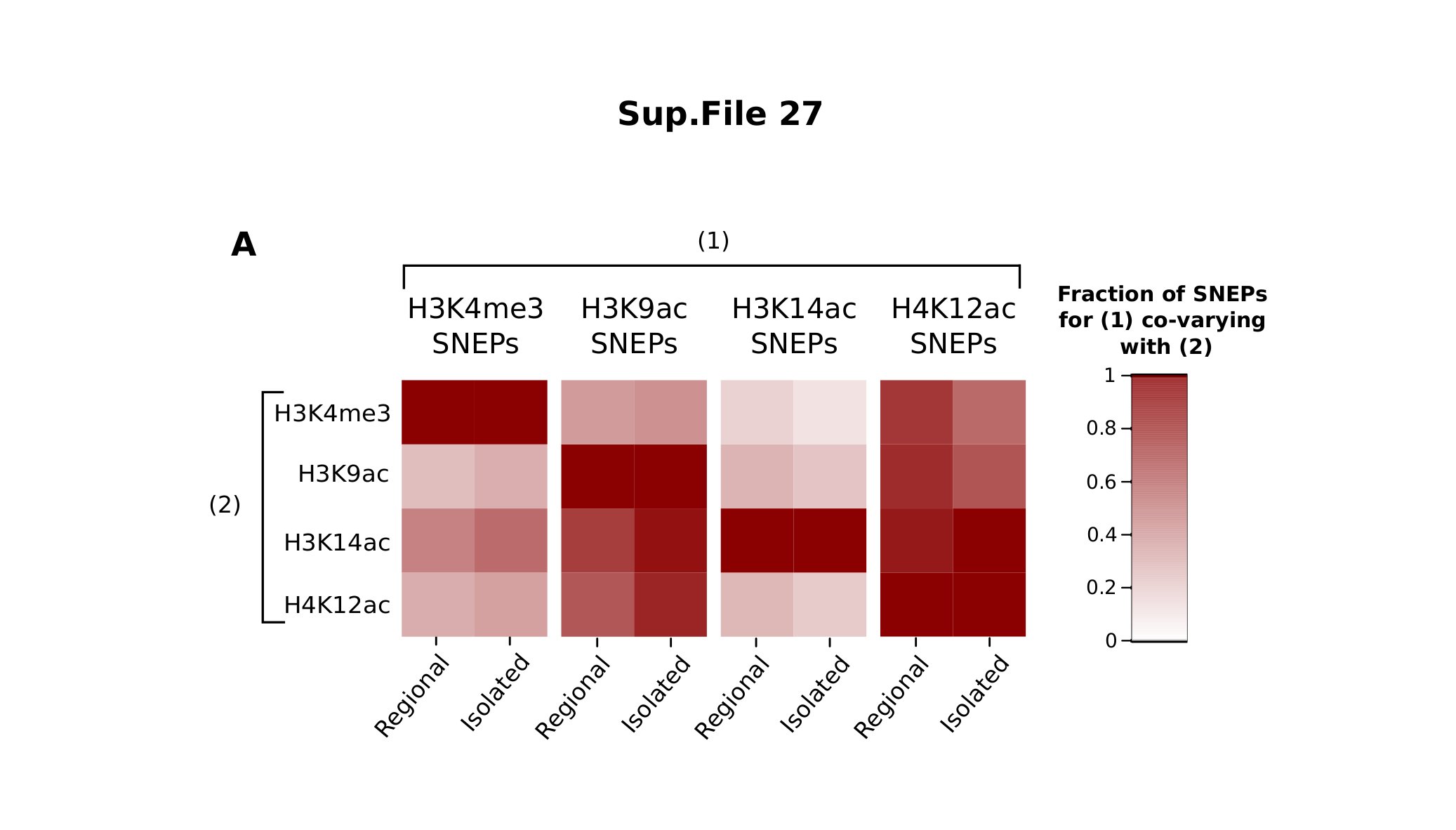

Supplement: Additional file 27: — Similar co-variation of histone marks at isolated vs. regional SNEPs. H3K4me3 SNEPs were termed ‘isolated’ when both flanking nucleosomes did not contain an H3K4me3 SNEP. All others were termed ‘regional’. The same definition was applied to SNEPs of other marks. On each set of nucleosomes (those corresponding to regional and those corresponding to isolated SNEPs for mark (1)), co-variation was quantified as in Fig. 8C, by computing the fraction of BY–RM isolated or regional SNEPs of mark (1) that showed synergistic and significant BY–RM differences in mark (2). [file 13072_2015_19_MOESM27_ESM.jpeg]

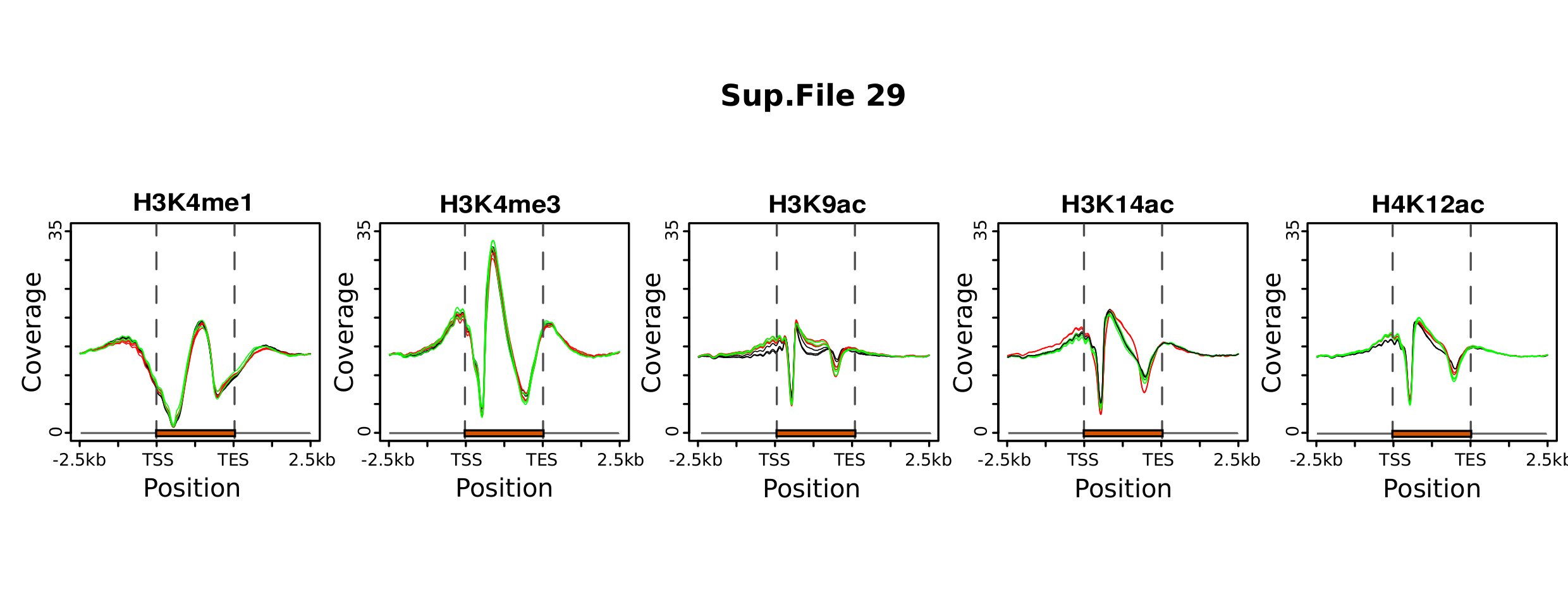

Supplement: Additional file 29: — ChIP coverage profiles of the indicated marks along an average gene for each sample (in per-million reads, normalized and averaged across replicates). [file 13072_2015_19_MOESM29_ESM.jpeg]
